# Towards precision in the diagnostic profiling of patients: leveraging symptom dynamics as a clinical characterisation dimension in the assessment of major depressive disorder

**DOI:** 10.1192/bjp.2024.19

**Published:** 2024-05

**Authors:** Omid V. Ebrahimi, Denny Borsboom, Ria H. A. Hoekstra, Sacha Epskamp, Edoardo G. Ostinelli, Jojanneke A. Bastiaansen, Andrea Cipriani

**Affiliations:** Department of Experimental Psychology, University of Oxford, Oxford, UK; and Department of Psychology , University of Oslo, Oslo, Norway; Department of Psychology, University of Amsterdam, Amsterdam, The Netherlands; Department of Psychology, National University of Singapore, Singapore, Singapore; Department of Psychiatry, University of Oxford, Oxford, UK; Oxford Precision Psychiatry Laboratory, NIHR Oxford Health Biomedical Research Centre, Oxford, UK; and Oxford Health NHS Foundation Trust, Warneford Hospital, Oxford, UK; Department of Psychiatry, University of Oxford, Oxford, UK; Oxford Precision Psychiatry Laboratory, NIHR Oxford Health Biomedical Research Centre, Oxford, UK; and Oxford Health NHS Foundation Trust, Warneford Hospital, Oxford, UK.; Interdisciplinary Center Psychopathology and Emotion Regulation, Department of Psychiatry, University of Groningen, University Medical Center Groningen, Groningen, The Netherlands; and Friesland Mental Health Care Services, Leeuwarden, The Netherlands.

**Keywords:** Depression, individual differences, symptom relationship patterns, personalised assessment, precision psychiatry

## Abstract

**Background:**

International guidelines present overall symptom severity as the key dimension for clinical characterisation of major depressive disorder (MDD). However, differences may reside within severity levels related to how symptoms interact in an individual patient, called symptom dynamics.

**Aims:**

To investigate these individual differences by estimating the proportion of patients that display differences in their symptom dynamics while sharing the same overall symptom severity.

**Method:**

Participants with MDD (*n* = 73; mean age 34.6 years, s.d. = 13.1; 56.2% female) rated their baseline symptom severity using the Inventory for Depressive Symptomatology Self-Report (IDS-SR). Momentary indicators for depressive symptoms were then collected through ecological momentary assessments five times per day for 28 days; 8395 observations were conducted (average per person: 115; s.d. = 16.8). Each participant's symptom dynamics were estimated using person-specific dynamic network models. Individual differences in these symptom relationship patterns in groups of participants sharing the same symptom severity levels were estimated using individual network invariance tests. Subsequently, the overall proportion of participants that displayed differential symptom dynamics while sharing the same symptom severity was calculated. A supplementary simulation study was conducted to investigate the accuracy of our methodology against false-positive results.

**Results:**

Differential symptom dynamics were identified across 63.0% (95% bootstrapped CI 41.0–82.1) of participants within the same severity group. The average false detection of individual differences was 2.2%.

**Conclusions:**

The majority of participants within the same depressive symptom severity group displayed differential symptom dynamics. Examining symptom dynamics provides information about person-specific psychopathological expression beyond severity levels by revealing how symptoms aggravate each other over time. These results suggest that symptom dynamics may be a promising new dimension for clinical characterisation, warranting replication in independent samples. To inform personalised treatment planning, a next step concerns linking different symptom relationship patterns to treatment response and clinical course, including patterns related to spontaneous recovery and forms of disorder progression.

Major depressive disorder (MDD) is the second leading cause of disability worldwide.^1^ Affecting over 350 million individuals globally, MDD is the most commonly diagnosed mental disorder in clinical practice.^1,2^ Although accurate assessment is an integral part of treatment, differences have been found across patients in the number of ways symptoms can be combined to satisfy the MDD diagnostic criteria.^3,4^ Accordingly, it has been argued that additional clinical characterisation is needed to more precisely understand the psychopathology of the individual patient and to personalise treatments.^5,6^ International clinical guidelines focus on overall symptom severity as the key criterion for treatment selection.^6–9^ This is problematic as additional layers of individual differences in psychopathological expression may be present within the same level of severity.^10^ We propose that this layer does not concern the number or severity of symptoms, but rather how symptoms interact with each other. This relates to the concept of symptom dynamics (or symptom relationship patterns), denoting how symptoms interact with one another over time in a given patient.^11,12^ This concept has been largely underrecognised in the mental health field, even though it has been shown that symptom relationship patterns may actively contribute to the expression of and any changes in the psychiatric condition.^11–16^ Two individuals with MDD of the same symptom severity may therefore exhibit substantially different patterns of interaction between their symptoms. Information about these patterns can help identify specific features and differentiate patients displaying the same symptom severity from each other.^10^ However, the extent to which individual differences are present along this dimension of psychopathology in patients with the same diagnosis and severity remains uninvestigated. In this study, we aimed to examine the proportion of patients with MDD that display individual differences in their symptom dynamics while sharing the same overall symptom severity.

## Method

This study used data collected at four secondary care facilities in The Netherlands during the ZELF-i study, a randomised controlled trial investigating self-monitoring using ecological momentary assessment in MDD patients.^17^ The authors assert that all procedures contributing to this work comply with the ethical standards of the relevant national and institutional committees on human experimentation and with the Helsinki Declaration of 1975, as revised in 2008. All procedures involving patients were approved by the Medical Ethical Committee of the University Medical Center Groningen (reference: 2015/530).

### Participants and procedure

Participants were recruited between 2016 and 2018 by clinicians who screened them for eligibility using DSM-IV.^18^ All participants: (a) were out-patients aged between 18–65 years; (b) had a current primary diagnosis of major depressive disorder (MDD) and indication for treatment of depression; (c) spoke Dutch; and (d) provided written informed consent. Exclusion criteria were: (a) presence of psychotic or (hypo)manic symptoms (i.e. any form of bipolar disorder); (b) need for urgent care (i.e. acute suicidality); and (c) inability to follow research procedures owing to the presence of intellectual disability or significant visual or hearing impairments. The participants received travel reimbursements and an additional €10 as compensation for internet usage if they used their own smartphones during the study. No additional compensation was provided for participation. Among the 110 participants who provided daily measurements in the ZELF-i study, 74 (67.3%) met the criteria of sharing a symptom severity score with at least one other participant and were waiting to start treatment, rendering them eligible for the present study.

### Measurements

#### Depressive symptom severity

Overall symptom severity at baseline was assessed using the self-reported Inventory of Depressive Symptomatology (IDS-SR).^19^ The IDS-SR is a 30-item instrument querying about the past 7 days, scored on a 4-point scale (0–3), with higher scores reflecting greater severity; total score 0–13: no depression; 14–21: mild depression; 22–30: moderate depression; 31–38: severe depression; 39 or more: very severe depression.^20^ The internal consistency was good for this scale, with a Cronbach's *α* of 0.84.

#### Momentary assessment

Following an ecological momentary assessment (EMA) design, intensive repeated measurements were planned with a fixed 3 h sampling frequency 5 times per day over a 28-day period for each participant, yielding up to a maximum of 140 possible assessments per person. Momentary assessments were conducted via links to a questionnaire on a secure website sent to each participant's smartphone; those without smartphones were lent one for the study.^17^ The links were sent via text messages, with the participants instructed to respond as soon as possible on receipt. The questionnaires were open for 30 min before the links expired.

All momentary assessment items mapping onto depressive symptomatology were included in this study. Specifically, (a) depressed mood was measured by the items feeling down and feeling cheerful (reversed); (b) anhedonia by feeling indifferent, listless and enthusiastic (reversed); (c) appetite change as reported deviations in hunger from one's stable average (i.e. being more or less hungry than usual); (d) restlessness by feeling calm (reversed), stressed and relaxed (reversed); (e) irritability by feeling irritated; and (f) lethargy with reports of being tired and energetic (reversed) (the items are listed in Supplementary Material 3, available at https://dx.doi.org/10.1192/bjp.2024.19). Items were rated on a visual analogue scale with scores ranging from 0 (not at all) to 100 (very much). Mean scores were used for symptoms with multiple affective indicators to retain all variables on a 0–100 scale. The items adhered to guidelines for dynamic assessment in EMA studies, being brief and unambiguous (measuring one state per item), present in the ESM Item Repository and worded to assess momentary states, to avoid recall bias.^21–23^

### Statistical analyses

Statistical analyses were performed in R (version 4.2.2 on macOS).

#### Dynamic network analysis

The graphical vector autoregressive model (GVAR) in the ‘graphicalVAR’ (version: 0.3.3) and ‘psychonetrics’ (version: 0.11.15) R packages were used to estimate regularised and unregularised network models respectively, yielding person-specific dynamic network models that identify symptom relationship patterns in each participant.^24,25^ The regularised network models incorporate the least absolute shrinkage and selection operator (LASSO) and use the Bayesian information criterion (BIC) to identify the best fitting model.^24^ Visualised networks are based on regularised models to facilitate sparsity and more easily portray the most important edges.^24^ In estimating individual differences in symptom dynamics, raw edge weights from unregularised network models were obtained to yield unbiased estimates (detailed below). To facilitate sensitivity and to address missing data, we followed recommendations using the Kalman filter with LASSO regularisation in the regularised models and full information maximum likelihood (FIML) in unregularised models.^26^ We further followed these simulation recommendations advising the inclusion of a maximum of six nodes with sample sizes around 75 to 100 time points per person to optimally recover the person-specific network structures.^26^ Following the stationarity assumption of GVAR models, all variables were investigated for linear, quadratic and cubic trends. Simulation studies show no difference between detrending all variables versus a specific variable if any trend is present.^27^ Accordingly, all variables were detrended for consistency.

The GVAR model produces two types of network per individual: a temporal and a contemporaneous.^24,25^ Each of these person-specific networks includes the depressive symptoms as variables in the network (nodes), with the lines between the nodes (edges) revealing the statistical relationship between them. These relationships may be positive or negative. They encode deviations from person-specific means, reflecting how higher levels on a variable compared with the person's average are associated with increases or decreases in other variables compared with the person's average.^12,24,25^ The temporal network encodes effects forward in time, with its directed edges portrayed as arrows. These temporal edges are regression coefficients representing a node's associated strength of impact on another node at a consecutive time point, while controlling for all other variables in the network.^24,25^ These time-lagged edges are referred to as Granger causal,^28^ reflecting satisfaction of the temporal criterion of causality, yielding information about which node temporally precedes another in the system. In this study employing a lag-1 GVAR model with 3 h between assessments, edges in the temporal network reflect a symptom's associated strength of impact on another 3 h later. The contemporaneous network embodies partial correlations to display the unique relationships between all nodes in the network after accounting for the temporal effects.^24,25^ In the dynamic network literature, these edges may be interpreted as dynamics that are faster than those captured in the lag-1 temporal model,^27^ reflecting the relationship between symptoms inside a 3 h time window in the present study.

#### Individual differences in depressive symptom dynamics

We used the individual network invariance test (INIT)^29^ to investigate our research question about differential symptom relationship patterns (i.e. differences in the edge weights) in groups of MDD participants sharing the same overall symptom severity (IDS-SR scores). INIT is a specialised test developed to inspect differences between networks.^29^ This test compares two models within the groups of participants matched on symptom severity: one model in which all edges in each of the person-specific network models are freely estimated (where symptom relations are different across participants) and a contrasting model in which all edges in these networks are constrained to be equal (where there are no differences in symptom relations). Simulation studies have identified the Akaike information criterion (AIC) to be the most sensitive criterion to identify the presence (or absence) of such differences between network structures, with lower AIC values reflecting the best fitting among the contrasting ‘no difference’ (homogeneous) versus difference (heterogeneous) models.^29^ In sample sizes similar to those in the present study (i.e. approximately 100 responses per individual) and with six nodes present, the INIT test has revealed optimal sensitivity to detect individual differences when constraints are placed on both the temporal and contemporaneous edges.^29^ The INIT weighs differences in temporal versus contemporaneous edges equally, and further takes into account differences in the strength versus the absence/presence of edges. Additional mathematical details of the INIT method are available elsewhere.^29^ We chose the test as it is conservative with respect to difference detection, minimising the risk of overestimation, as it more easily detects no difference than differences in symptom dynamics (i.e. greater chance of a type II than a type I error). In summary, in a comparison of, for example, three individuals with MDD matching on severity levels (e.g. all three with a symptom severity score of 47), the INIT test reveals whether these three individuals display no difference in their symptom dynamics and thus whether they all can be represented by one (i.e. the same) network model, or whether they display differential symptom relations and therefore three models (one for each) must be estimated to represent their unique symptom dynamics. In a sensitivity analysis using a logistic regression, we checked whether individual difference detection was related to symptom severity levels (IDS-SR total scores range: 15–51) and severity group size (range: 2–6).

To obtain the proportion of MDD participants who shared the same overall symptom severity and displayed individual differences in their symptom dynamics, the total number of participants matched on symptom severity who were identified as displaying differences in their symptom dynamics was divided by the total number of participants in the sample. To construct a 95% confidence interval around the estimated proportion, this calculation procedure was repeated 10 000 times following random draws with replacement of the severity groups.^30^

#### Simulation inspecting the robustness of the INIT method in our sample

To investigate the extent to which the INIT method was susceptible to false detection of individual differences given the specific conditions of our study (e.g. number of individuals in each severity group, precise number of responses and missing values per participant), we conducted a supplementary simulation (full details in Supplementary Material 1). This procedure reflects obtaining the expected proportion of individual differences under a simulated null scenario where no such individual differences should be present.^29^

## Results

Demographic information for the sample is presented in Table 1. Among the 74 eligible participants sharing a depression symptom severity (IDS-SR) score with at least one other participant, the time series of 1 of the 74 (participant 11) violated the assumption of stationarity, yielding a final sample of 73 individuals. We observed 23 severity levels (i.e. 23 IDS-SR severity values) shared by a minimum of 2 participants, forming 23 groups of participants with MDD matched on overall symptom severity. Each group included between 2 and 6 matched participants. The most frequent IDS-SR severity category in the sample was ‘severe’ depression, consisting of 7 severity levels (IDS-SR scores of 31–35 and 37–38). All eligible participants provided enough data (i.e. a minimum of 75 completed assessments) to be included in the analysis.^26^ The mean response rate to the dynamic momentary assessments was 115 of 140 (82.1%; s.d. = 16.8) completed assessments per person.

**Table 1:** Demographic characteristics of the sample (*n* = 73)

Characteristic	Mean (s.d.) or *n* (%)
Age, years	
Mean	34.57 (13.12)
Range	18–64
Gender	
Female	41 (56.16%)
Male	32 (43.84%)
Educational level	
None, primary school, or lower secondary	16 (21.92%)
High school or lower vocational education	43 (58.90%)
Higher vocational or university degree	14 (19.18%)
Employment	
Employed	46 (63.01%)
Unemployed	27 (36.99%)
Living alone	
Yes	15 (20.55%)
No	58 (79.45%)
Any previous treatment for depression	
Yes	35 (47.95%)
No	38 (52.05%)

The INIT identified individual differences in symptom dynamics across 46 of the 73 participants, revealing differential symptom relationship patterns in 63.0% (95% bootstrapped CI 41.0–82.1) of MDD participants matched on overall symptom severity.

In the simulation study, we found the average false detection of individual differences with our method to be 2.2%. In the sensitivity analysis, we found both IDS-SR severity scores (logit = 0.61, s.e. = 0.40, *p* = 0.13) and size of severity group (logit = 0.004, s.e. = 0.43, *p* = 0.93) to be unrelated to individual difference detection in symptom dynamics.

The symptom relationship patterns of all 73 participants in this study are provided in Supplementary Material 2, illustrating differences and similarities in symptom interactions across the matched participants in each of the 23 severity groups.

**Fig. 1:**
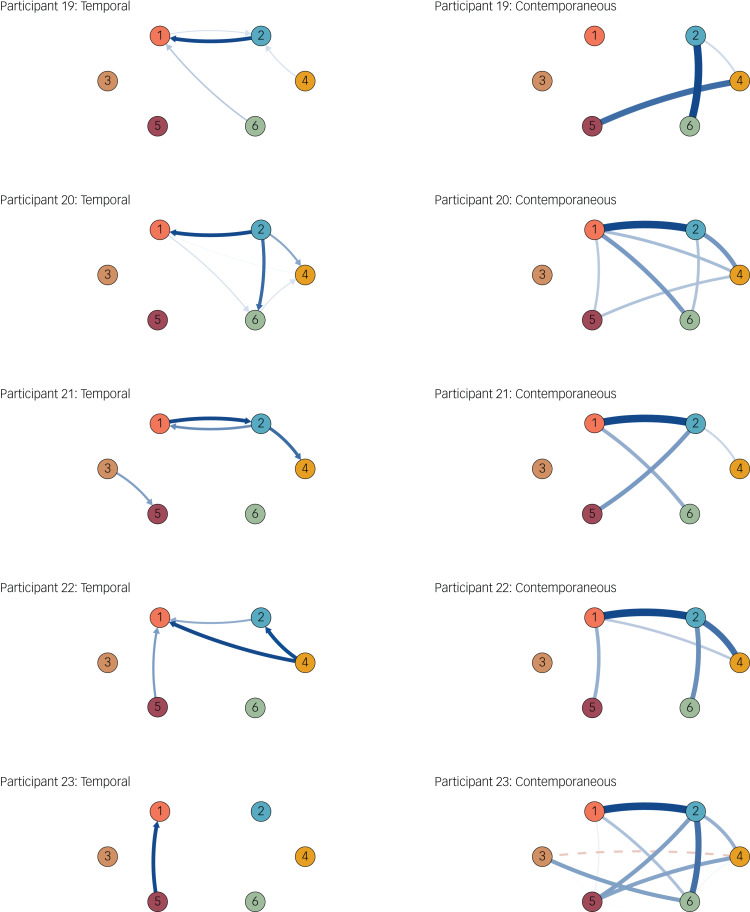
Symptom dynamics of the five participants with a score of 31 on the Inventory for Depressive Symptomatology Self-Report. Solid lines show positive relationships and the dashed line shows the only negative relationship. Node descriptions: 1, Anhedonia; 2, Depressed mood; 3, Appetite change; 4, Restlessness; 5, Irritability; 6, Lethargy.

To demonstrate these individual differences in symptom dynamics, we present the results for the first available symptom severity level in the most frequently observed severity category in our sample (i.e. an IDS-SR score of 31, for ‘severe depression’). We further describe participants matching on demographic characteristics. Five participants had an IDS-SR severity score of 31 (Fig. 1). Participants 19, 20 and 23 were female, in a relationship, aged 23–24 years and had had a high-school education. The temporal networks for these three show that although lethargy temporally precedes an increase in anhedonia for participant 19 (i.e. reflecting that greater lethargy is related to anhedonia 3 h later for this individual), this is opposite for participant 20, where anhedonia (along with depressed mood) is related to greater lethargy. Similarly, among other dynamics, the temporal pattern of restlessness is the opposite for these participants. Deviating even more strongly from the two aforementioned participants, participant 23's temporal symptom dynamics show it is an increase in irritability that is related to increases in anhedonia at the next time point. Moreover, a vicious cycle between depressed mood and anhedonia, where these symptoms increase and amplify one another, is present for some participants (e.g. participant 19), but not participant 23.

An inspection of the contemporaneous networks in Fig. 1 also reveals differential symptom relations for these participants, among which we can see that for participants 20 and 23, while these individuals experience anhedonia, this is associated with an increase in the experience of depressed mood, whereas this deleterious relationship between the core two of symptoms of depression is absent for participant 19. Similarly, in contrast to participants 19 and 20, where this relationship was absent, appetite change was associated with decreases in restlessness for participant 23.

## Discussion

In this paper, we presented the concept of symptom dynamics as a clinical characterisation dimension and showed that these symptom interactions vary in the majority of individuals with depression who share the same symptom severity. These results are consistent with previous studies identifying variability in symptom relationship patterns.^31,32^ We found such individual differences also to be present when individuals share the same overall symptom severity, highlighting that symptom dynamics can serve as a characteristic to differentiate between the psychopathological expression of patients with the same diagnosis and severity classification.

In the MDD participants who displayed the same depressive symptom severity in the present sample, we identified differences in how symptoms temporally precede and are associated with the elevation of other symptoms. This is consistent with research finding differential symptom interactions in individuals with schizophrenia.^33^ These identified differences suggest that general psychoeducation on the level of the diagnosis presented to patients is limited in precisely describing the key symptoms that play a dominant role in worsening the condition for a specific individual. Capturing symptom dynamics therefore gives patients an opportunity to obtain a person-specific understanding of their experience together with their clinician.^33,34^

Our findings also highlight the importance of temporal monitoring of depressive symptoms from the perspective that an understanding of how symptoms fluctuate and interact over time can provide information about the formation of the disorder.^35^ Previous studies have highlighted the active role of symptom dynamics in worsening mental health conditions, where it has been found that not all symptoms of depression should be considered equal with respect to their reach and strength of impact on the aggravation of additional depressive symptomatology over time.^12,36^ Moreover, symptoms identified to be more strongly connected with other symptoms were found to be more likely to predict the onset of future depression compared with less strongly connected symptoms.^37^

Studies have also found differential treatment effects on specific symptoms of depression^38–40^ and that change in one symptom during the course of psychotherapy is highly dependent on change in other symptoms.^41^ Accordingly, the presence of differences in symptom relationship patterns across patients is likely to have implications for treatment. This is because recent studies have found indirect (i.e. secondary) effects on specific symptoms via changes first occurring in other symptoms directly influenced by treatments.^42,43^ This reflects that an understanding of the symptom relationship patterns in a particular patient presents opportunities for personalised treatment. A recent trial investigated this by obtaining the symptom relationship patterns of different individuals with eating disorders through ecological momentary assessments, where clinicians thereafter used this information to match patients to specific evidence-based psychotherapy modules aimed at targeting symptoms with the strongest and highest number of connections to other symptoms in each patient.^44^ This personalised therapy trial presented promising preliminary results related to the acceptability of dynamic assessment and the personalisation procedure, the feasibility with respect to low drop-out rates, and found treatment effects to be retained up to a year.^44^

Moreover, recent studies have shown that individuals are able to describe the dynamic patterns between their experienced symptoms, and that a greater number of feedback loops (i.e. mutually reinforcing relations) between their symptoms is associated with increased symptom frequencies.^45,46^ This highlights the utility of symptom dynamics in clinical assessment settings and its potential for obtaining greater precision in the psychopathological characterisation and treatment monitoring of patients.

### Limitations and strengths

This study has several limitations. The proportion of individual differences in symptom dynamics is likely to have been underestimated, given the method's conservativeness.^29^ We chose this conservative method to present the minimum likely proportion of individual differences in symptom dynamics. As the underlying GVAR model captures linear dynamics at a lag-1 level, a general limitation concerns this model's ability to capture non-linear dynamics and possible effects operating on different timescales and lags,^12^ prompting future investigations to examine these topics through complementary modelling frameworks.^32,47^ The degree to which momentary states can be accurately related to symptoms necessitates further investigation. Four symptoms of depression (concentration difficulties, worthlessness, sleep disturbance and suicidal ideation) were unavailable in our data and thus not investigated. The participants received feedback on provided data during the EMA period. Although this was shown to not be associated with symptom changes,^48^ feedback during the assessment period may have influenced the findings, and thus be a limitation. As DSM-5 introduced new specifiers, the screening of patients for MDD using DSM-IV may have led to a broader subgroup of patients. Furthermore, evidence suggests that unipolar depression may in some individuals convert to bipolar depression over time.^49^ The short duration of investigation (i.e. 28 days) and the exclusion of individuals with bipolar disorder, however, makes this less likely to have influenced our findings. Although this study includes a large number of observations per person, the study's sample size for between-participant comparison is small, resulting in the availability of 23 of the 84 possible severity levels on the IDS-SR, and thus reduced precision (i.e. wider confidence intervals) in the individual differences estimate in this study.

This study also has strengths. Having obtained 115 completed measurements on average per participant (i.e. 8395 observations), this is a large EMA study with respect to information on person-specific dynamics. The high measurement adherence rate (average of 82%) adds to the quality of the data.^50^ The study recruited individuals diagnosed by mental health professionals in real-world clinical settings, adding to its proximity and generalisability to the clinic.^51^ Further adding to its clinical proximity, this study followed recommended guidelines in assessing participants’ overall symptom severity.^7^ As a next step, investigating individual differences in symptom dynamics when individuals share the same item-level scores could present added opportunities for clinical characterisation and treatment personalisation.

### Future directions

Findings from this study highlight areas for future research. One question concerns how differential symptom dynamics may relate to the evolution of the disorder, referring to how a disorder will progress on its own over time. Different types of symptom connections were identified across individuals with MDD in the present study. A naturally ensuing question is whether certain patterns of symptom interactions relate differently to changes in the disorder state, such as clinical profiles in which spontaneous recovery occurs and critical cases where the disorder worsens over time. This would translate into the clinical question of whether it is possible to know, for a specific patient, whether it is best to prioritise a watchful waiting approach (e.g. when the individual is optimally recovering) or a more rapid initiation of treatment to avoid worsening.

Systematic investigations of symptom relationship patterns may also provide information on whether different clusters of diagnostic profiles deriving from symptom dynamics exist within diagnostic domains.^3,32^ This may be a promising line of research towards improving precision in the psychopathological assessment of patients.^47^ In the present study, we also found evidence for sizeable proportions of patients (i.e. 37%) that displayed similar symptom relationship patterns. The presence of shared profiles of symptom dynamics across patients could enable the identification of different risk factors, prognoses and treatment response patterns tied to the profiles.

Moreover, individual differences in symptom relationship patterns may occur not only in major depression, but also in other disorder domains. Findings from the eating disorders domain suggest that this may be the case.^52^ Accordingly, the extent to which individual differences in symptom dynamics are present across other disorder domains remains an open question. To facilitate the investigation of this topic, we openly provide all materials (code for person-specific network models, individual difference analysis and simulation setup) needed to research this topic in other substantive areas, found at the online repository of the Center for Open Science (https://osf.io/ygk84/). Finally, a next step concerns investigating whether an examination of changes in symptom relationship patterns during treatments can offer additional insight about the symptom-specific and indirect effects of psychological and psychopharmacological interventions.

## Supporting information

Ebrahimi et al. supplementary material 1 Ebrahimi et al. supplementary material

Ebrahimi et al. supplementary material 2 Ebrahimi et al. supplementary material

Ebrahimi et al. supplementary material 3 Ebrahimi et al. supplementary material

## Data Availability

Access to the data that support the findings of this study may be requested from the ZELF-i team (Dr Jojanneke Bastiaansen or Dr Harriëtte Riese). All code for the study has been uploaded to the online repository of the Center for Open Science (https://osf.io/ygk84/), where we also provide all materials (code for person-specific network models, individual difference analysis and simulation set-up) needed to research this topic in other substantive areas.
