# Supplementary material for: Towards precision in the diagnostic profiling of patients: leveraging symptom dynamics as a clinical characterisation dimension in the assessment of major depressive disorder
Source: Br J Psychiatry. 2024 May;224(5):157–63. doi: 10.1192/bjp.2024.19 (PMC11039556; doi:10.1192/bjp.2024.19)
Supplement: Ebrahimi et al. supplementary material 1 — Ebrahimi et al. supplementary material [file S0007125024000199sup001.pdf]

**Supplemental Material 1: Supplementary Simulation**

To investigate the robustness of our obtained estimate of the proportion of individual differences in depressive symptom dynamics, we conducted a supplementary simulation study that matched the specific conditions of our original study. The Individual Network Invariance Test (INIT) has thus far been tested in an extensive simulation study to compare the network structure of *pairs* of individuals[1]. In our study, we applied INIT in a novel manner to compare *groups* of individuals based on their severity score. As we cannot assume INIT has perfect specificity, we set up a simulation study to investigate the false positive rate when comparing the network structure of groups of individuals with the same severity score.

**Simulation setup**

We used our original data containing symptom dynamics of 73 MDD outpatients measured 5 times a day over a period of 28 days ($N_t$ = up to 140 per person; average completed assessments per person = 115) to estimate the average within-person contemporaneous and temporal network structures using a multilevel vector autoregressive (VAR) approach[2,3,4]. The resulting network structures are revealed in Figure A1, hereafter referred to as the null model. We took the estimated parameters from these network structures to simulate homogeneous time-series data (i.e. simulated data with no individual differences in symptom dynamics across patients) using the *graphicalVARsim* function from the *graphicalVAR* package[4,5], with the R-code for the simulation setup provided in the online supplementary materials. This reflects that, in the simulation study, we use the network structure in Figure A1 as the true underlying network structure across all 73 participants in our simulated time-series data. To resemble the original data as close as possible, we replaced participants symptom dynamic measurements in the original data file with our generated homogeneous data values, keeping other characteristics of the data such as the number of missing values, the number of beeps, day of assessment, severity scores, and accordingly

severity groups fully intact. We refer to this dataset as the null-model data. Using this null-model dataset, we followed our original procedures to investigate the false positive rate for INIT when comparing groups. Thus, differences in group sizes' of number of matched patients fully represented the original procedure, as well as the number of time-series per individual and their missing values.

Following the procedure in our original study, we used unregularized edge weights that were obtained for each patient using the *'psychonetrics'* package in R[6,7]. Based on these network structures, INIT compares two models within the groups of matched patients: one model in which all edge weights in each person-specific network structure are freely estimated (i.e. individual differences in symptom relations and thus an estimation of one separate network model per person within the matched groups of patients) to a model in which all edge weights between the person-specific networks are constrained to be equal (i.e., no individual differences in the network structures (i.e. symptom relations) within the matched groups of patients). Using the AIC as a guidance for model fit[1], we determined which of these two models (i.e., unconstrained versus constrained) fits the data best within each severity level group. To get an indication of the false positive rate we repeated the above-described simulation procedure 100 times.

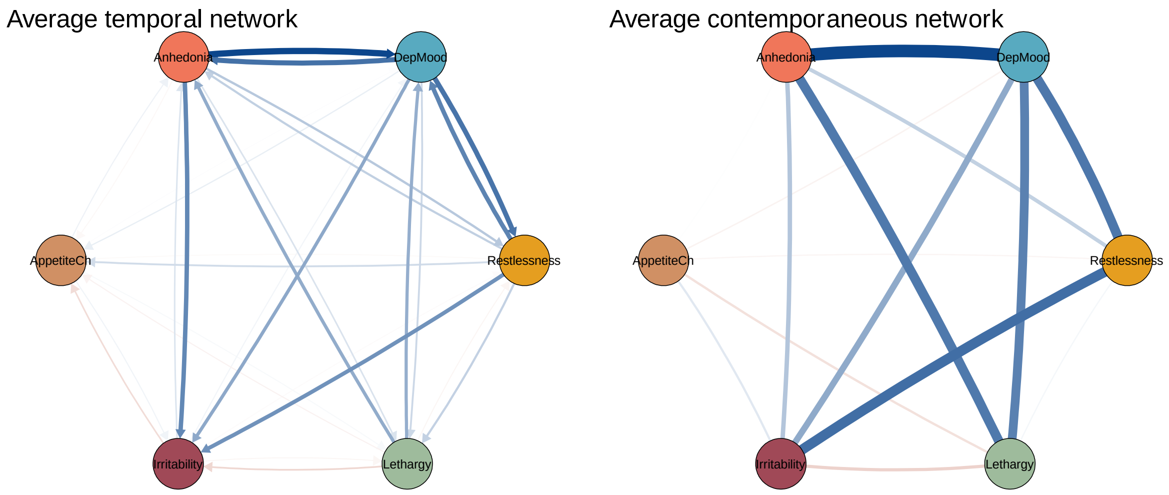

*Figure A1.* The left panel shows the average within-person temporal network. The right panel shows the average within-person contemporaneous network. Estimated parameter values from these networks were used to generate homogeneous time-series data (i.e. simulated data with no individual differences in symptom relations across patients).

**Simulation results**

The results of the simulation study revealed that under the null model (i.e., the homogenous model where no individual differences should be present), we would expect an average false positive rate of 2% (0.02±0.03; mean±SD). When calculating the proportion of individual differences as the percentage of individuals for which we found an individual difference model to fit the data better than the indifference model, we obtain a proportion of 2.22% (3.33%; SD) of individual differences on average when no such individual differences should be present. This means, when individual differences in symptom dynamics are absent, we would expect to wrongfully conclude individual differences to be present 2.22% of the times on average with a range of (0%-17.81%). Our obtained proportion estimate of individual differences in depressive symptom dynamics of 63.01% is considerably greater than this identified false positive rate, corroborating the robustness of this estimate in our study.

**Bootstrapping confidence intervals**

The individual difference estimate has been computed as the number of individuals for which a heterogeneous model was favoured (n = 46), based on the Individual Network Comparison Test (INIT), over the total number of individuals in the sample (n = 73) (i.e., 46/73*100 = 63.01%). To create a 95% confidence interval around this individual difference estimate, we used a bootstrap method[8].

The bootstrapping procedure is as follows: a data frame consisting of the results of INIT for each of the 23 severity cohorts and the number of participants in each of the severity

cohorts is created. We refer to this data frame as our sample. To bootstrap a confidence interval around the original 63.01% individual difference estimate, we randomly draw data from this sample with replacement in order to create a new sample. This means, in the new sample, some of the severity cohorts and their corresponding INIT result can be included multiple times. Based upon this resample, we calculate the resample individual differences. This is called the bootstrap estimate of our individual differences. We store this bootstrap estimate and repeat the process 10,000 times. Based upon these bootstrapped estimates, we can create a 95% bootstrapped confidence interval by taking the lower bound of 2.5% and the upper bound of 97.5% (as $\alpha$ = 0.05/2 = 0.025) of these estimates.

This yields a confidence interval of 40.98% and 82.05% around our 63.01% individual difference estimate. Of note, the present bootstrap interval is based on estimations of the INIT method. While the INIT method is the state-of-the-art technique for the (in)difference testing in network models, it is a conversative method that more easily detects indifference rather than differences in symptom dynamics (i.e. greater chance of type II than a type I error).

In summary, our obtained estimate of individual differences in the symptom dynamics of MDD patients matched on symptom severity, 63.01% (95% CI = [40.98, 82.05]), was substantially above the identified false positive rate for the detection of such individual differences (2.22%), with the individual differences in symptom relations across patients further likely to be even higher due to the conservativeness our method.

**REFERENCES**


1. Hoekstra RH, Borsboom D, McNally R, Epskamp S. Testing similarity in longitudinal networks: The Individual Network Invariance Test (INIT). In preparation 2022.

2. Bringmann LF, Vissers N, Wichers M, et al. A network approach to psychopathology: new insights into clinical longitudinal data. PLoS One 2013;8:e60188.

3. Epskamp, S., Deserno, M. K., Bringmann, L. F. & Veenman, M. mlVAR: Multi-level vector autoregression [Computer software manual] 2021a. (R package version 0.10).

4. Epskamp S, Waldorp LJ, Mõttus R, Borsboom D. The Gaussian Graphical Model in Cross-Sectional and Time-Series Data. Multivariate Behavioral Research 2018;53:453-80.

5. Epskamp, S. graphicalVAR: Graphical VAR for experience sampling data [Computer software manual] 2021b. (R package version 0.3).

6. Epskamp S. Psychometric network models from time-series and panel data. Psychometrika 2020;85:206-31.

7. Epskamp, S. psychonetrics: Structural equation modeling and confirmatory network analysis. [Computer software manual] 2021c. (R package version 0.10).

8. DiCiccio TJ, Efron B. Bootstrap confidence intervals. Statistical Science 1996;11:189-228.
