# Supplementary material for: Towards precision in the diagnostic profiling of patients: leveraging symptom dynamics as a clinical characterisation dimension in the assessment of major depressive disorder
Source: Br J Psychiatry. 2024 May;224(5):157–63. doi: 10.1192/bjp.2024.19 (PMC11039556; doi:10.1192/bjp.2024.19)

Symptom dynamics of MDD patients with IDS−SR score: 15

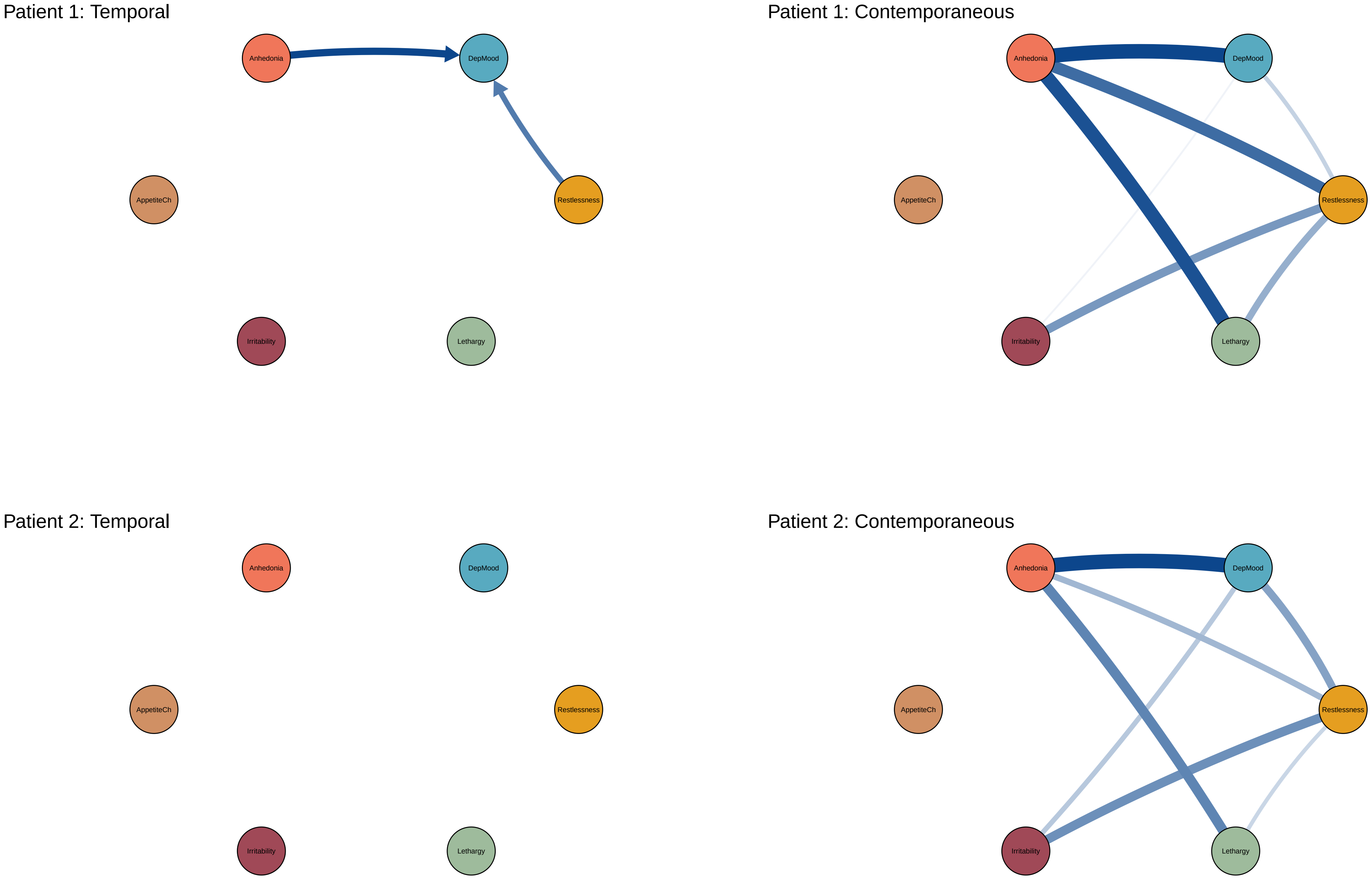

Symptom dynamics of MDD patients with IDS−SR score: 16

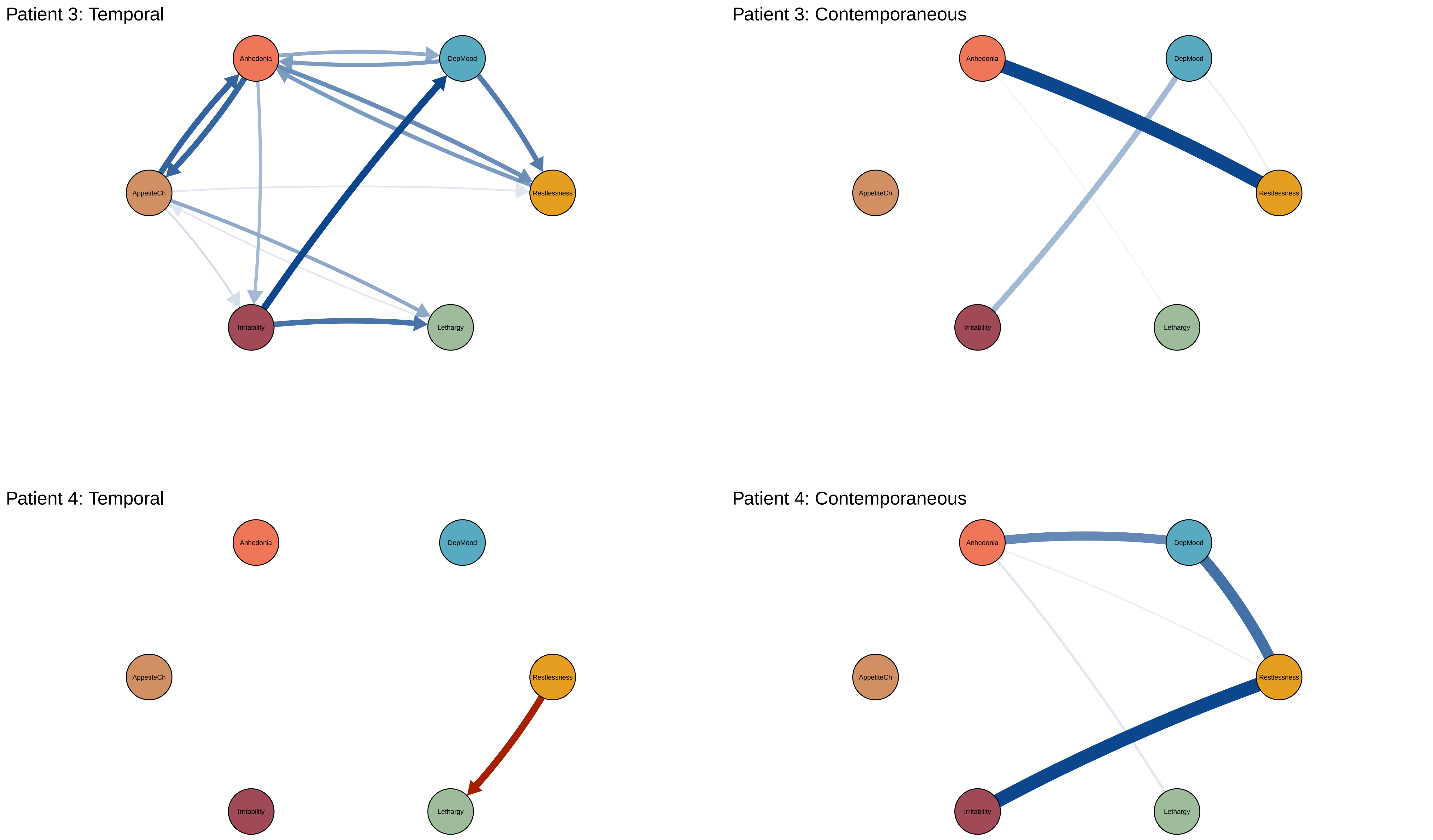

Symptom dynamics of MDD patients with IDS−SR score: 21

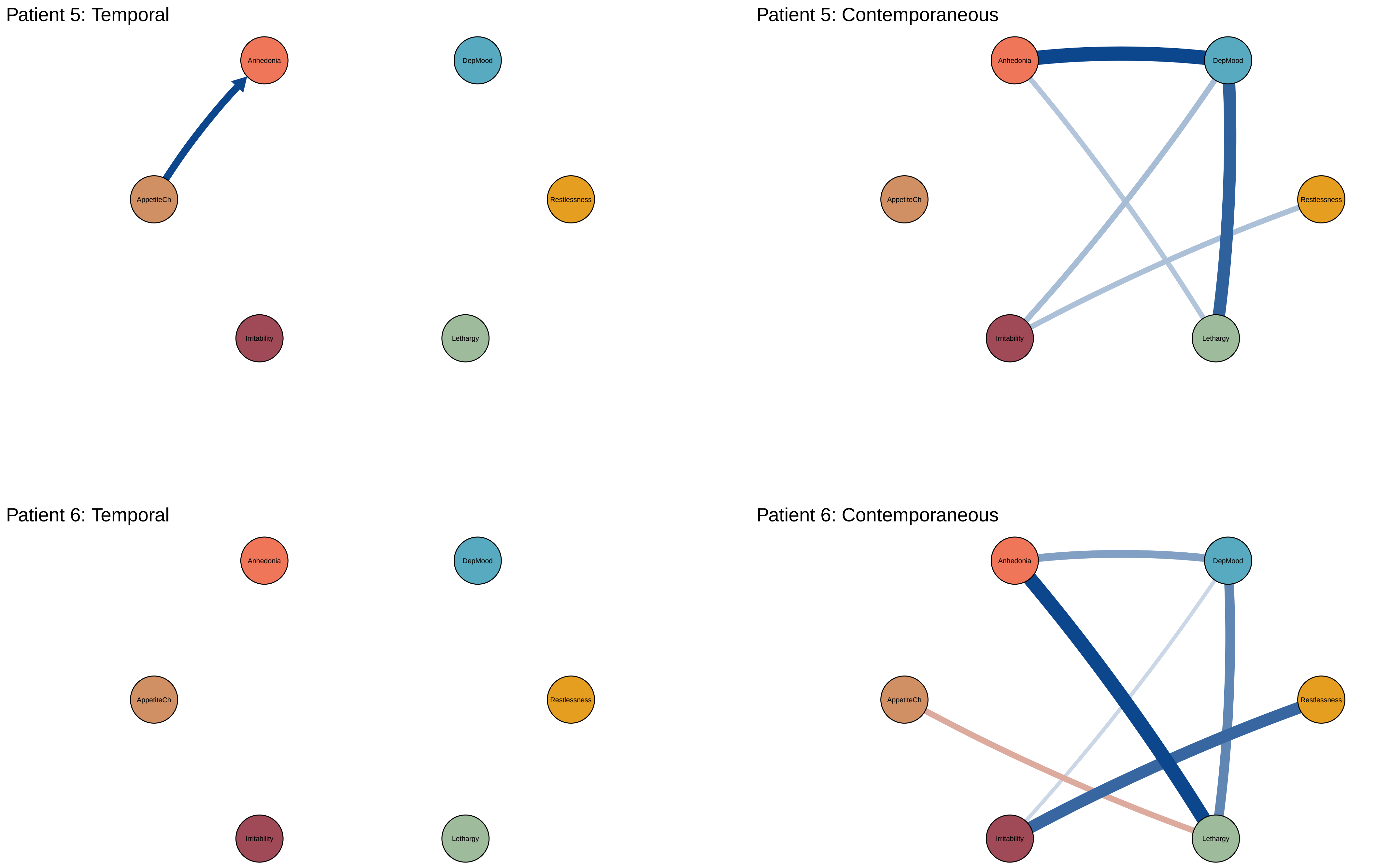

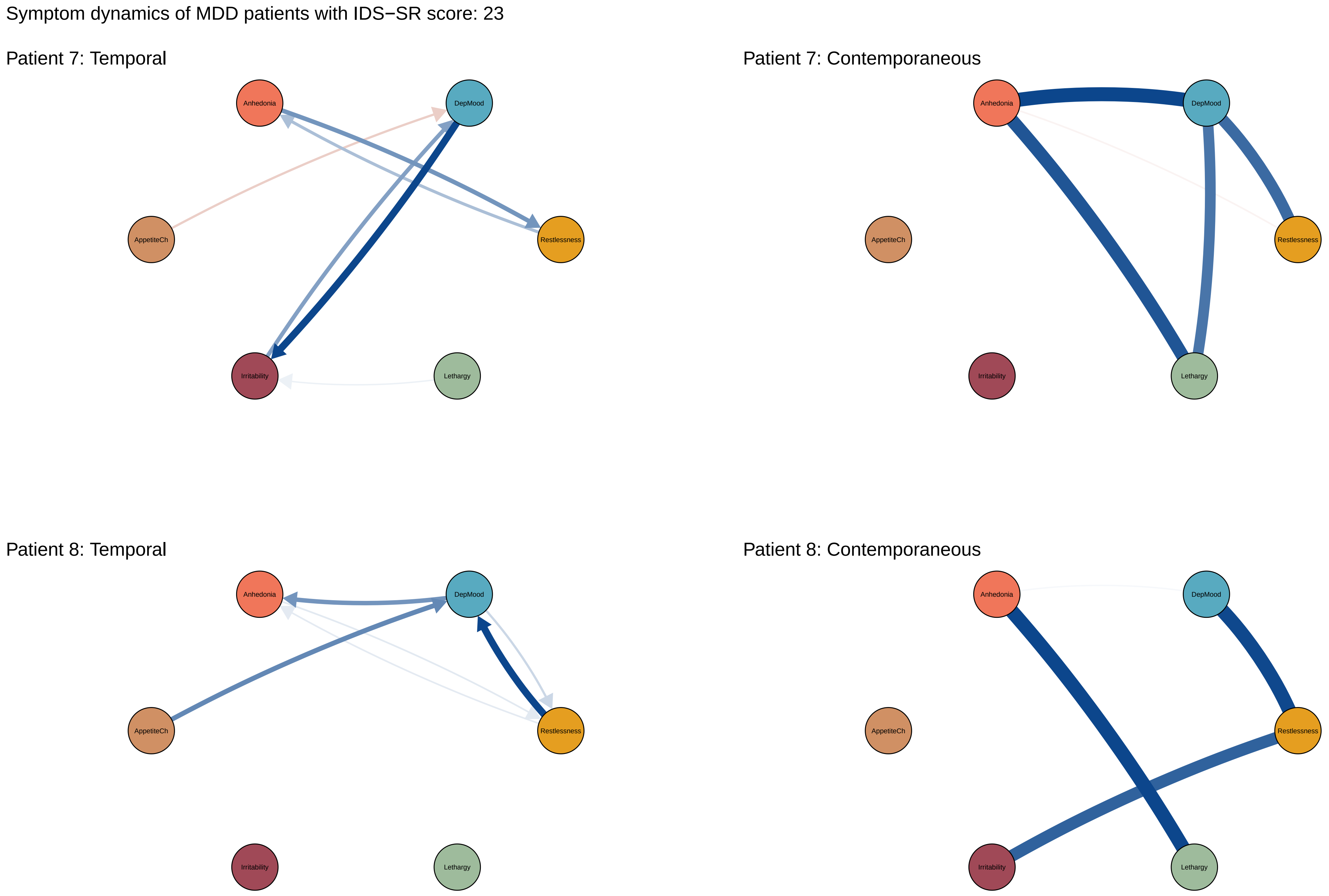
Symptom dynamics of MDD patients with IDS−SR score: 23
Patient 7: Temporal
Anhedonia
DepMood
AppetiteCh
Restlessness
Irritability
Lethargy
Patient 7: Contemporaneous
Anhedonia
DepMood
AppetiteCh
Restlessness
Irritability
Lethargy
Patient 8: Temporal
Anhedonia
DepMood
AppetiteCh
Restlessness
Irritability
Lethargy
Patient 8: Contemporaneous
Anhedonia
DepMood
AppetiteCh
Restlessness
Irritability
Lethargy

Symptom dynamics of MDD patients with IDS−SR score: 28

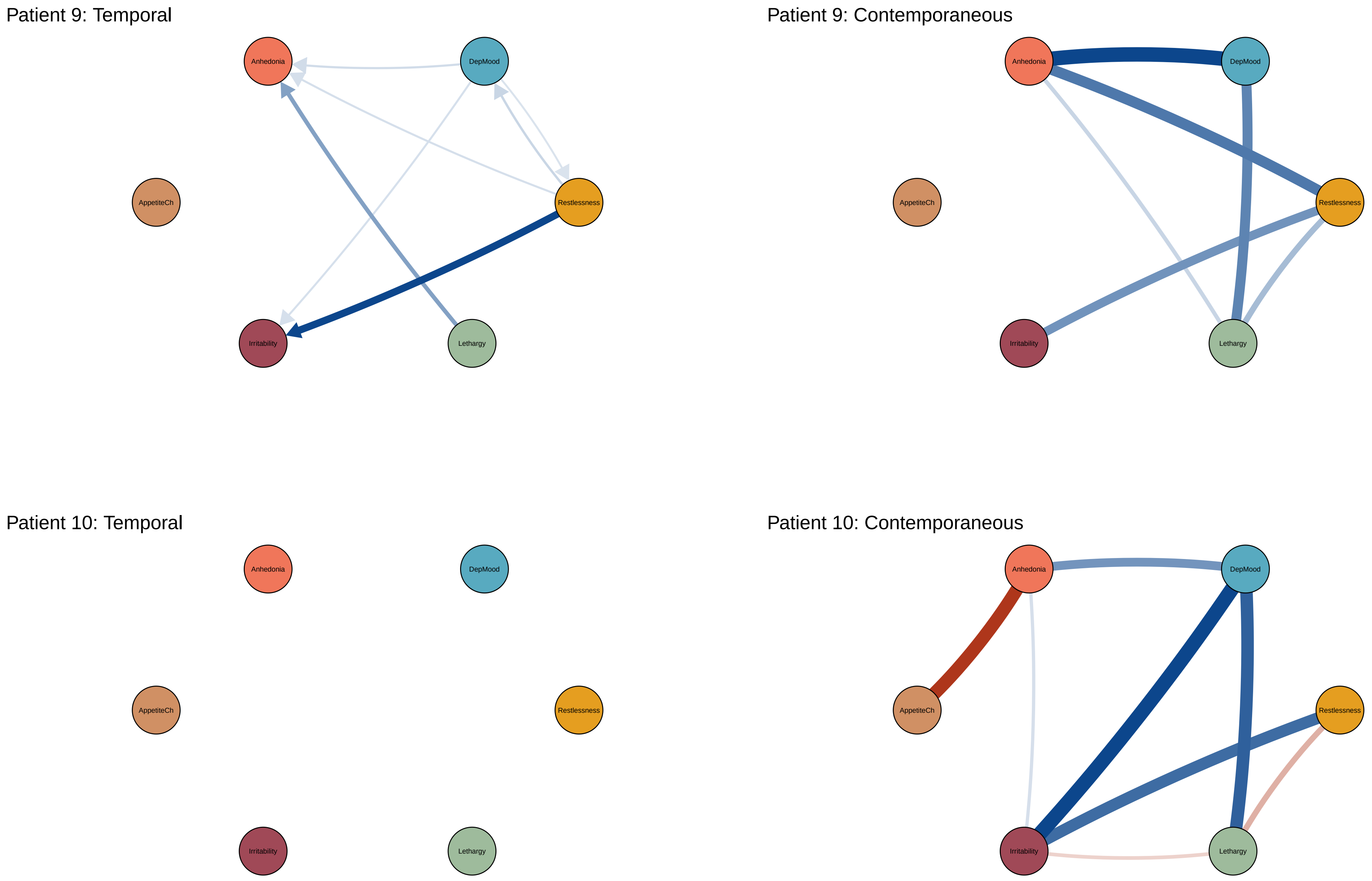

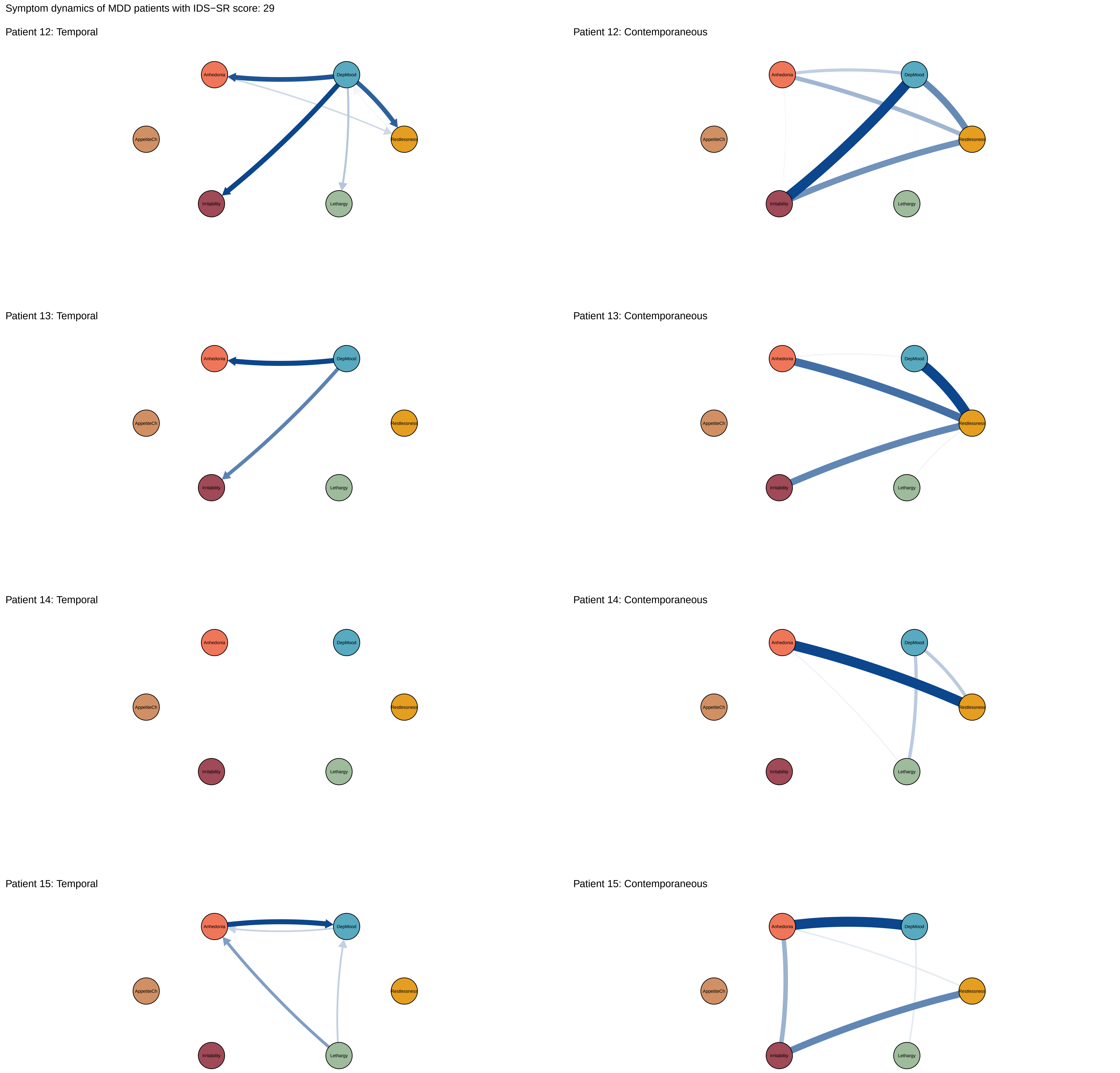
Symptom dynamics of MDD patients with IDS−SR score: 29
Patient 12: Temporal
Patient 12: Contemporaneous
Patient 13: Temporal
Patient 13: Contemporaneous
Patient 14: Temporal
Patient 14: Contemporaneous
Patient 15: Temporal
Patient 15: Contemporaneous
Anhedonia
DepMood
AppetiteCh
Restlessness
Irritability
Lethargy

Symptom dynamics of MDD patients with IDS−SR score: 30

Patient 16: Temporal

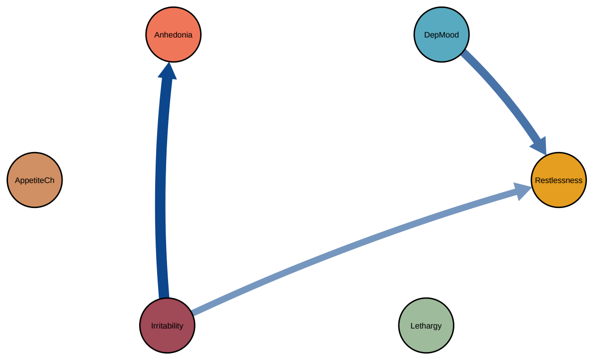

Patient 16: Contemporaneous

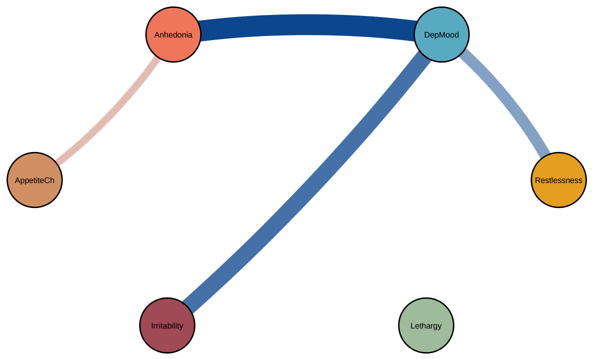

Patient 17: Temporal

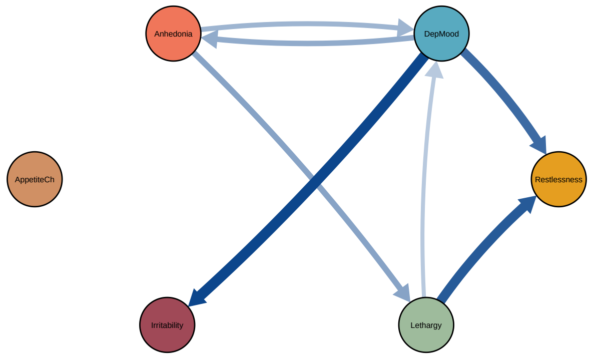

Patient 17: Contemporaneous

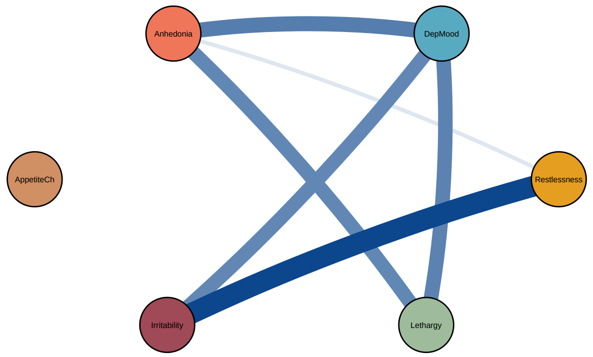

Patient 18: Temporal

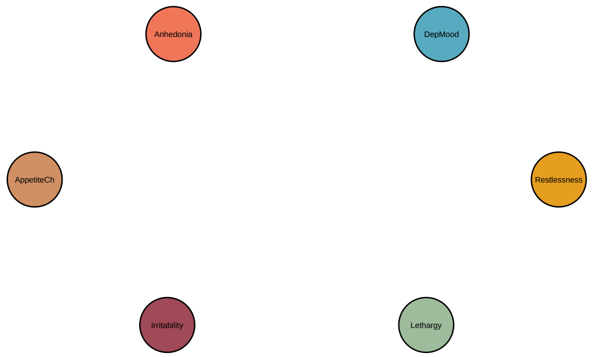

Patient 18: Contemporaneous

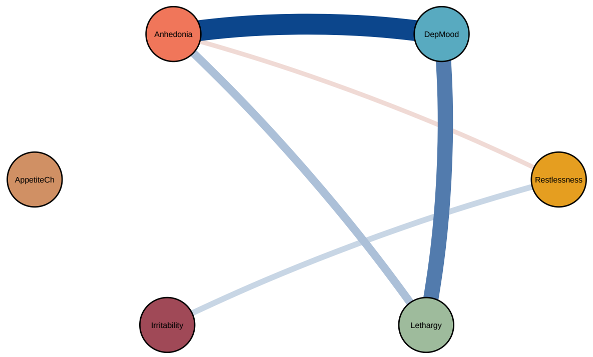

Symptom dynamics of MDD patients with IDS−SR score: 31

Patient 19: Temporal

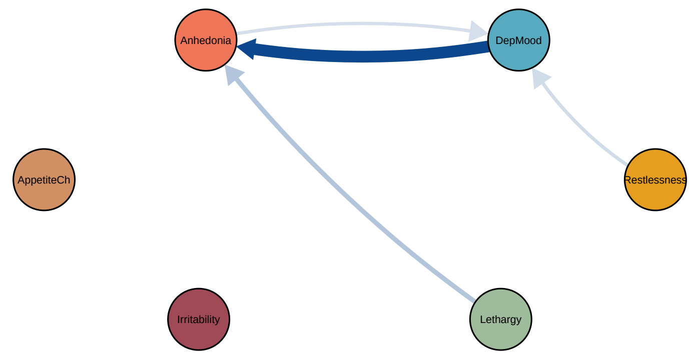

Patient 19: Contemporaneous

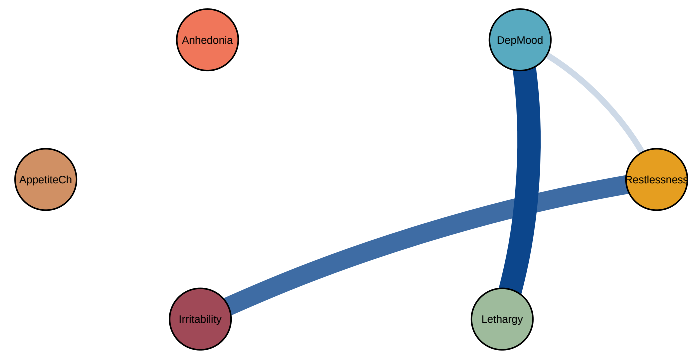

Patient 20: Temporal

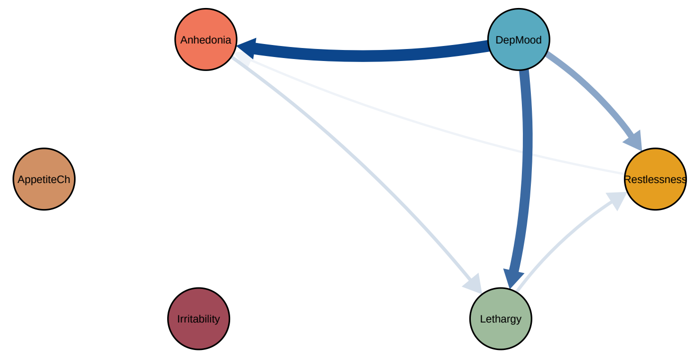

Patient 20: Contemporaneous

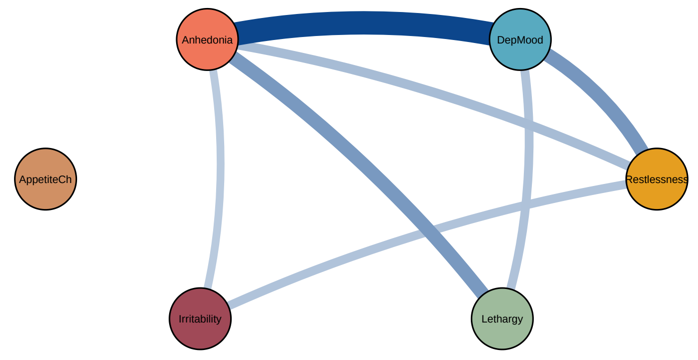

Patient 21: Temporal

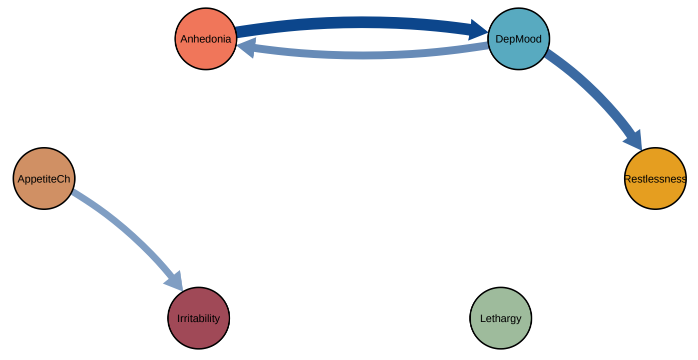

Patient 21: Contemporaneous

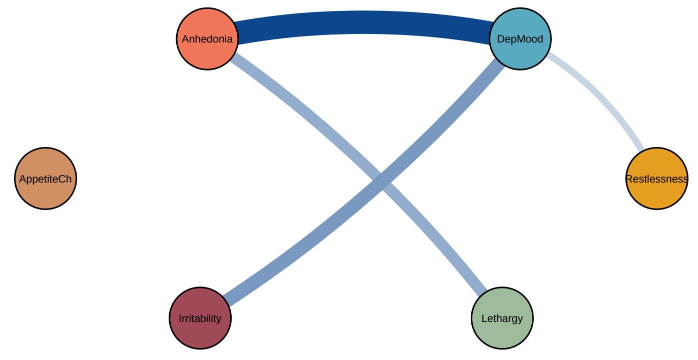

Patient 22: Temporal

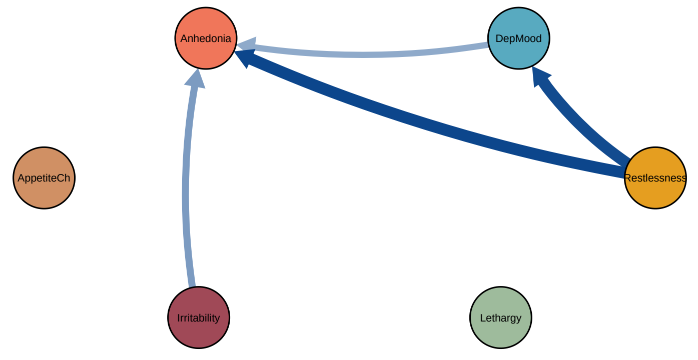

Patient 22: Contemporaneous

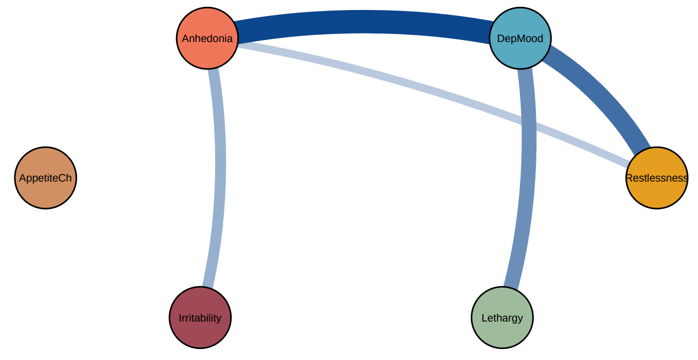

Patient 23: Temporal

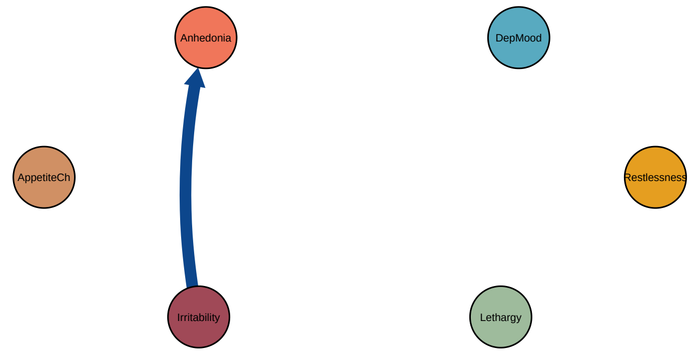

Patient 23: Contemporaneous

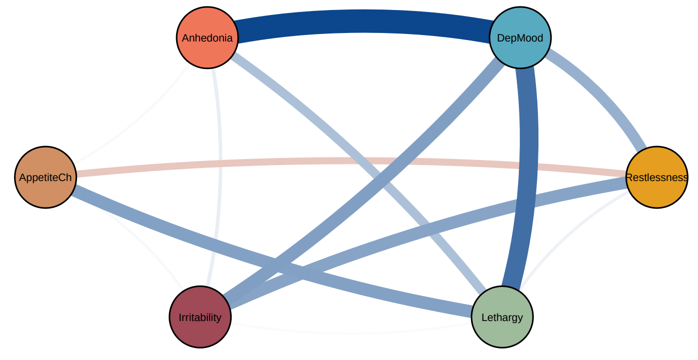

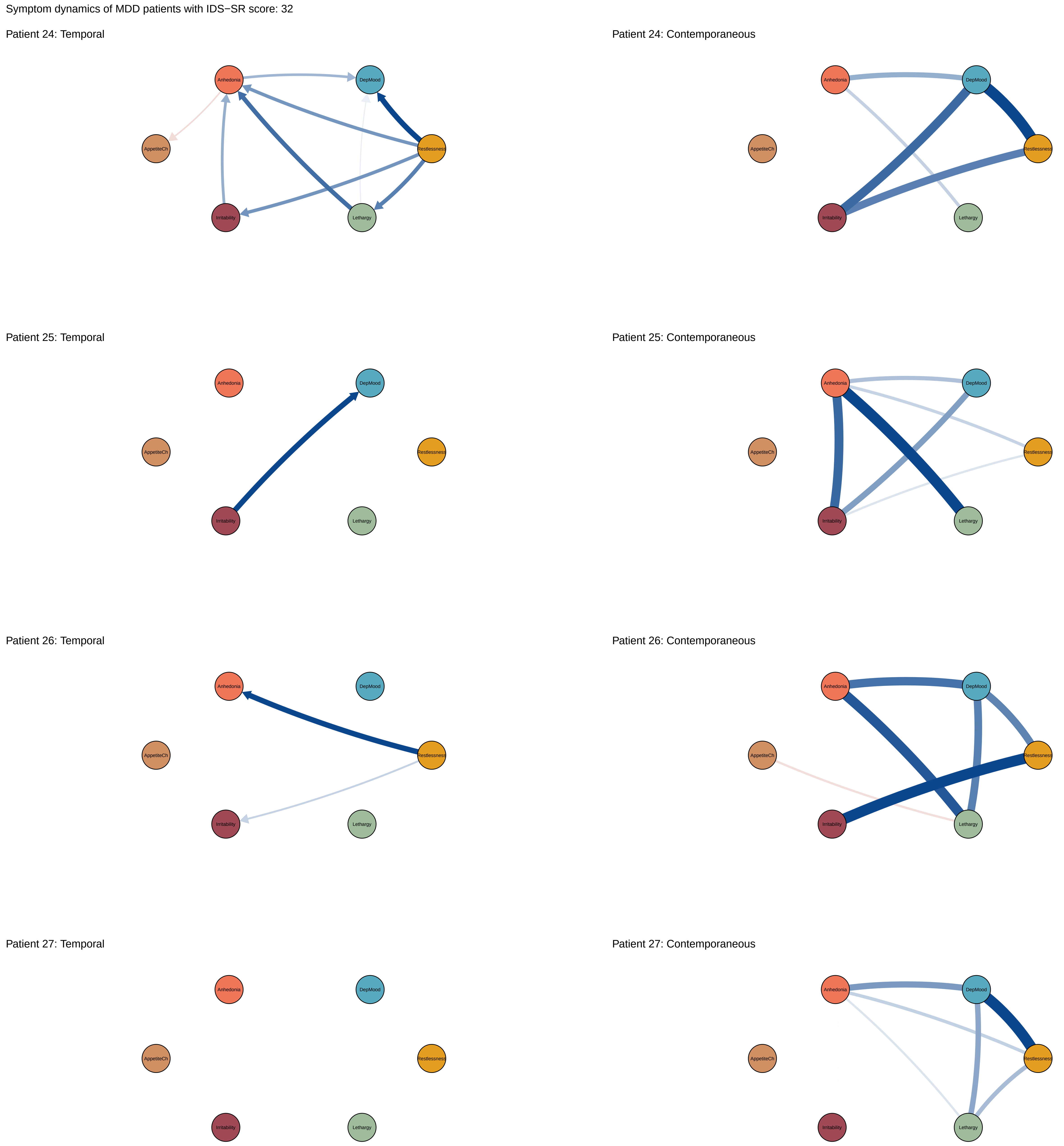
Symptom dynamics of MDD patients with IDS−SR score: 32
Patient 24: Temporal
Patient 24: Contemporaneous
Patient 25: Temporal
Patient 25: Contemporaneous
Patient 26: Temporal
Patient 26: Contemporaneous
Patient 27: Temporal
Patient 27: Contemporaneous
Anhedonia
DepMood
AppetiteCh
Restlessness
Irritability
Lethargy

Symptom dynamics of MDD patients with IDS−SR score: 33

Patient 28: Temporal

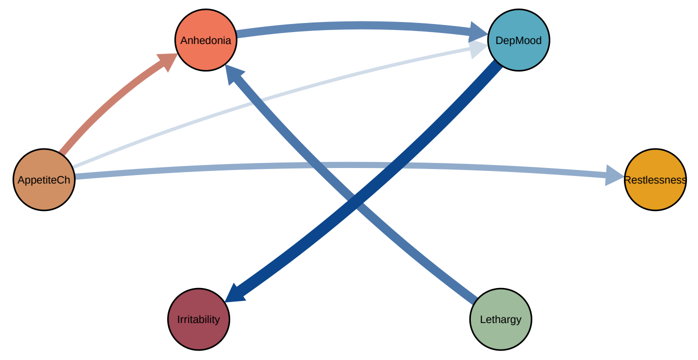

Patient 28: Contemporaneous

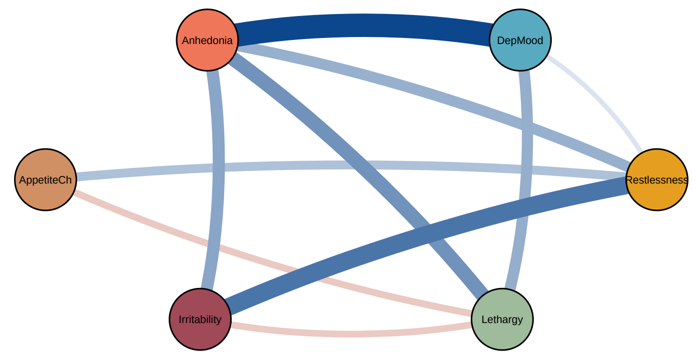

Patient 29: Temporal

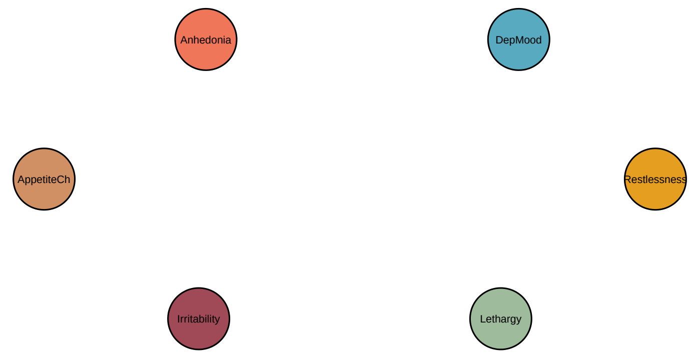

Patient 29: Contemporaneous

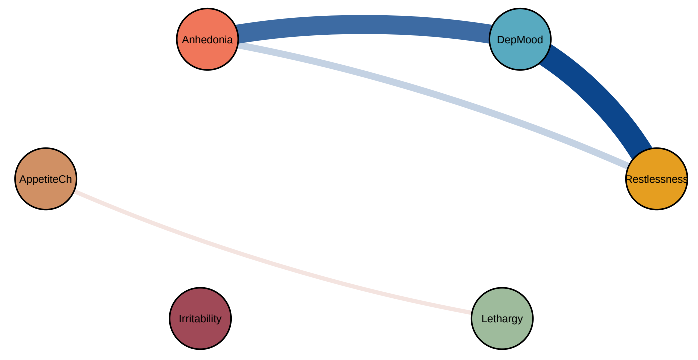

Patient 30: Temporal

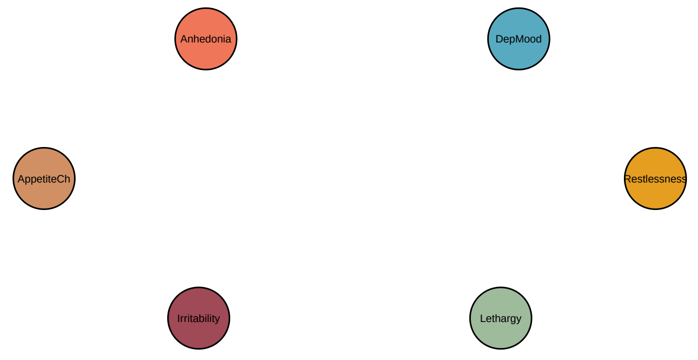

Patient 30: Contemporaneous

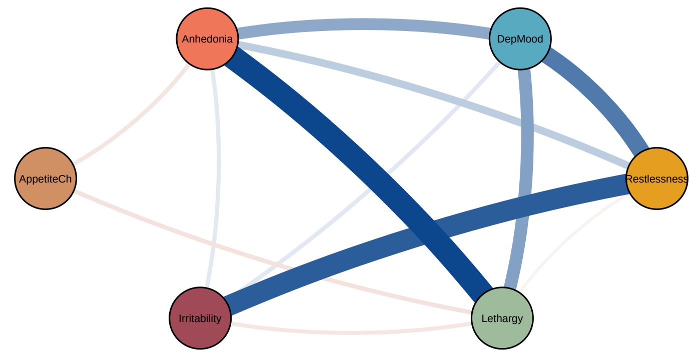

Patient 31: Temporal

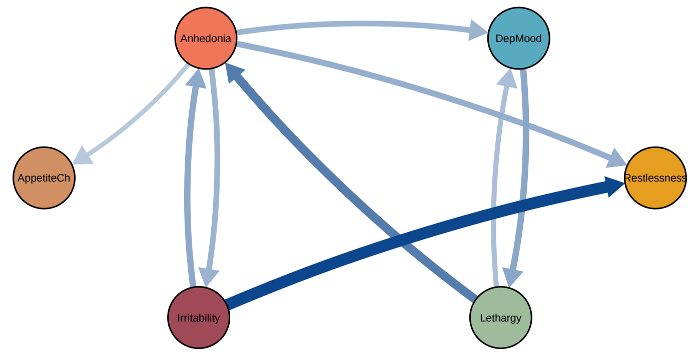

Patient 31: Contemporaneous

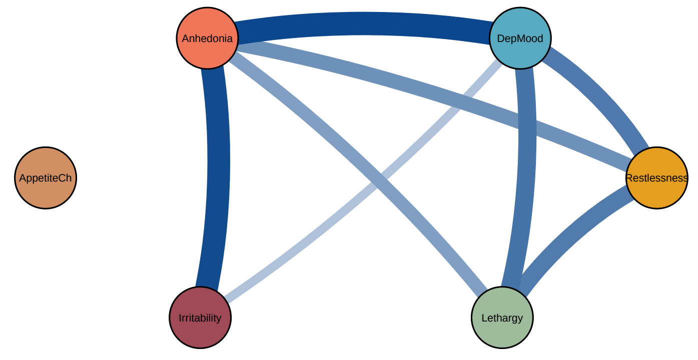

Patient 32: Temporal

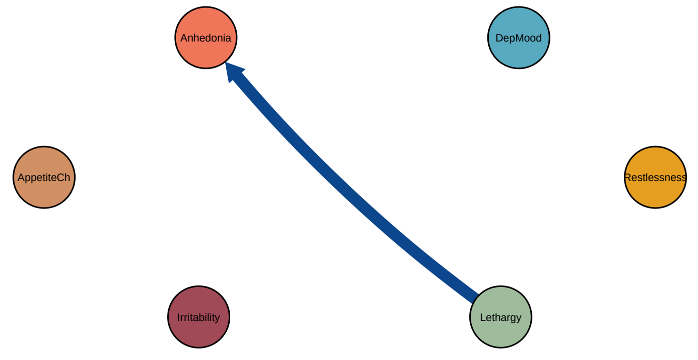

Patient 32: Contemporaneous

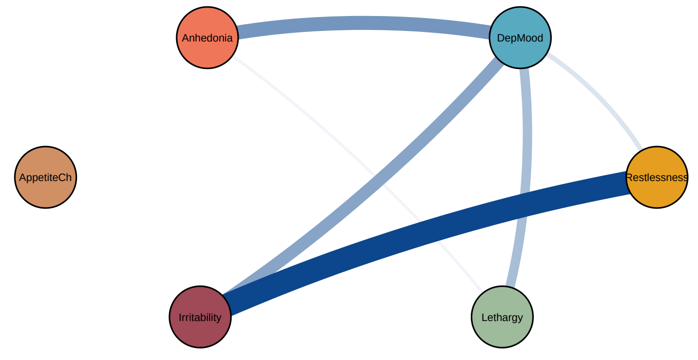

Symptom dynamics of MDD patients with IDS−SR score: 34

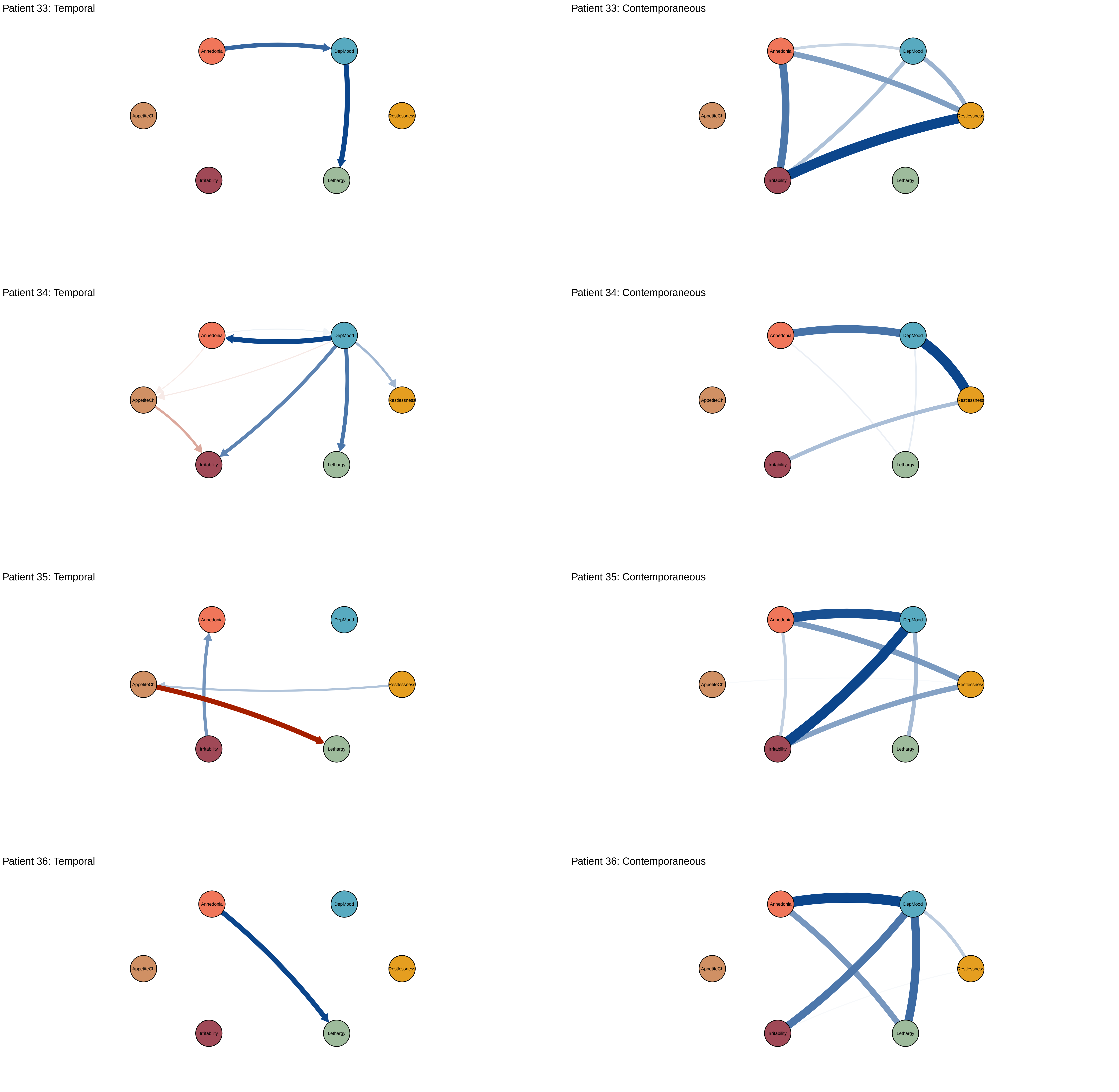

Symptom dynamics of MDD patients with IDS−SR score: 35

Patient 37: Temporal

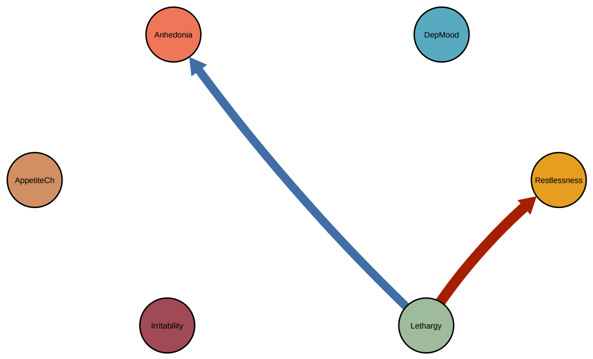

Patient 37: Contemporaneous

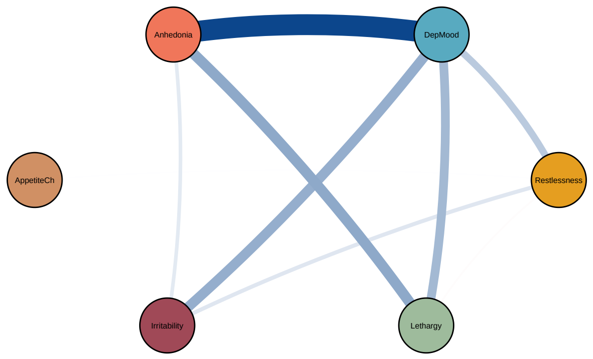

Patient 38: Temporal

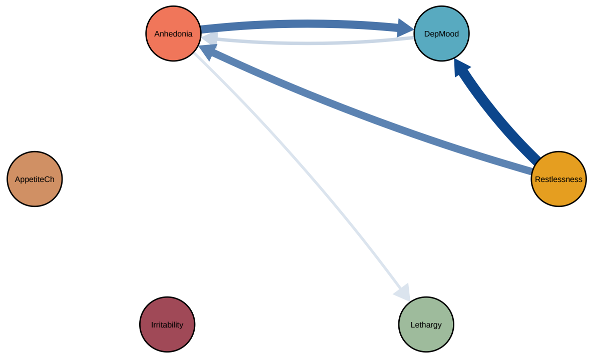

Patient 38: Contemporaneous

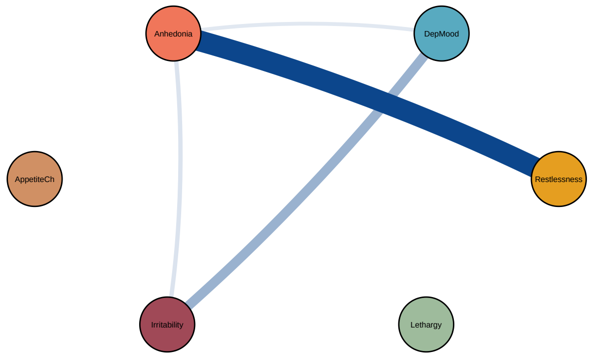

Patient 39: Temporal

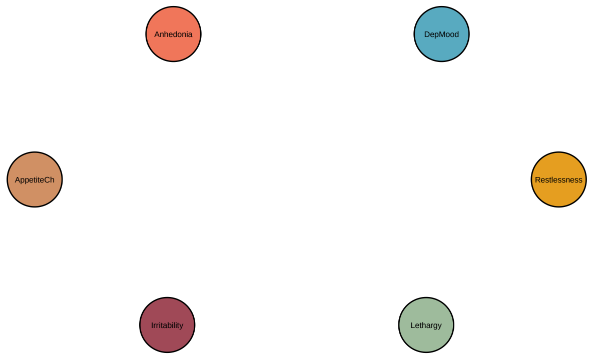

Patient 39: Contemporaneous

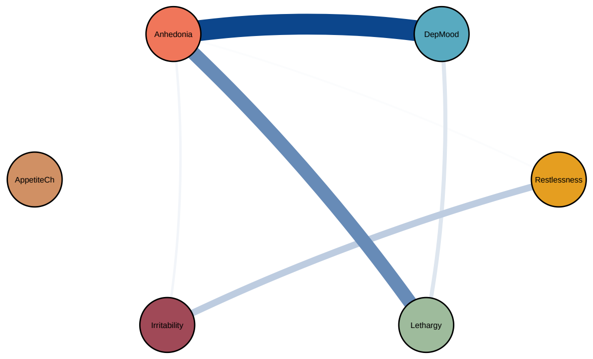

Symptom dynamics of MDD patients with IDS−SR score: 37

Patient 40: Temporal

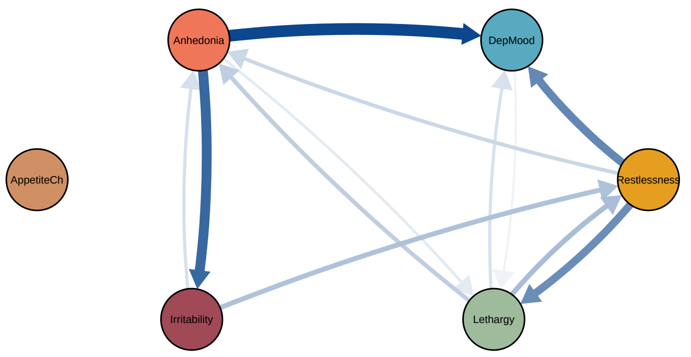

Patient 40: Contemporaneous

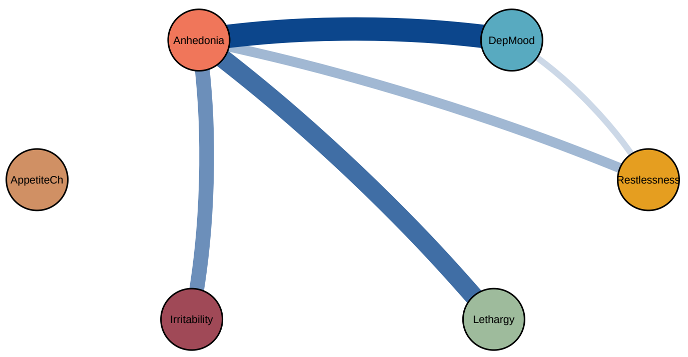

Patient 41: Temporal

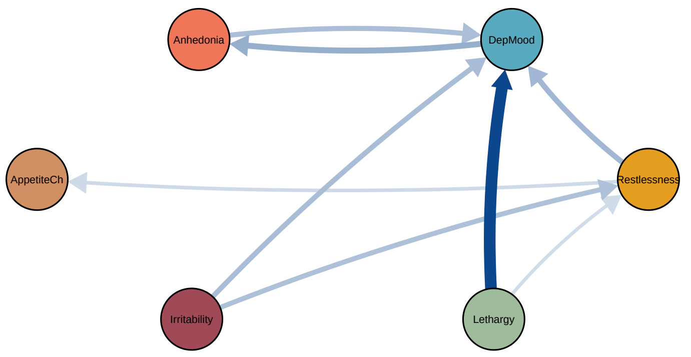

Patient 41: Contemporaneous

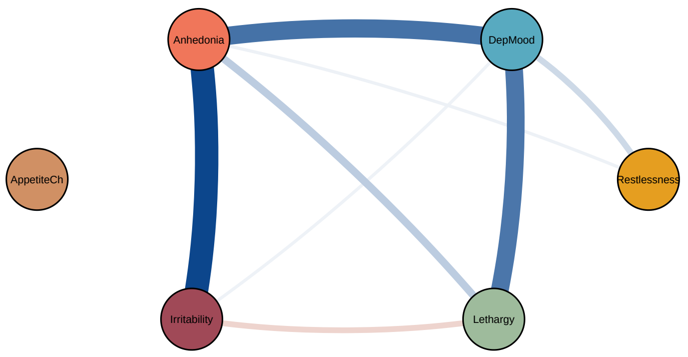

Patient 42: Temporal

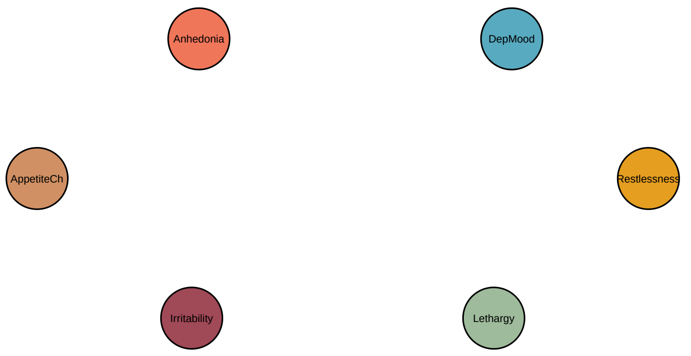

Patient 42: Contemporaneous

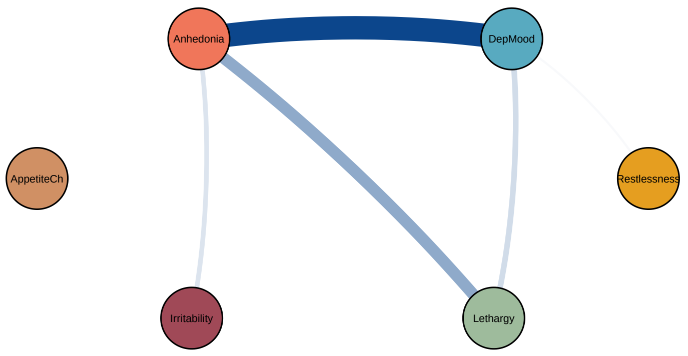

Patient 43: Temporal

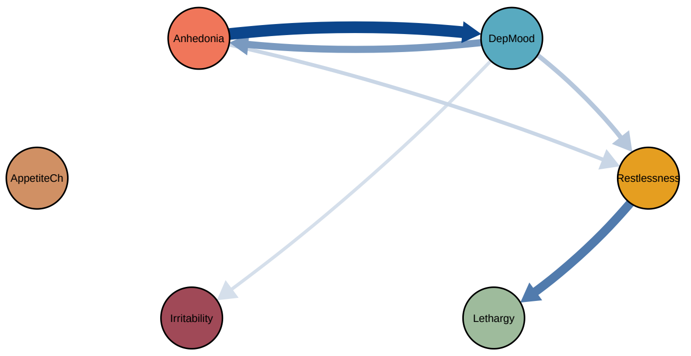

Patient 43: Contemporaneous

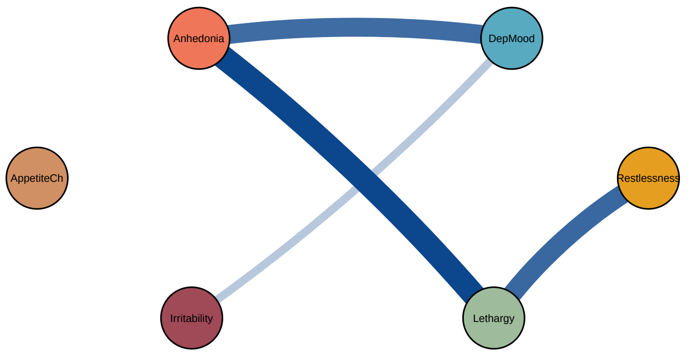

Patient 44: Temporal

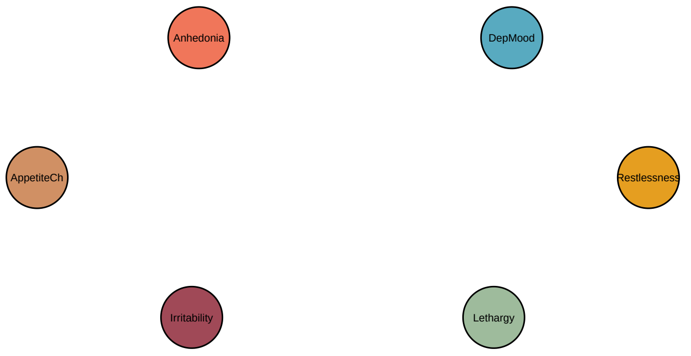

Patient 44: Contemporaneous

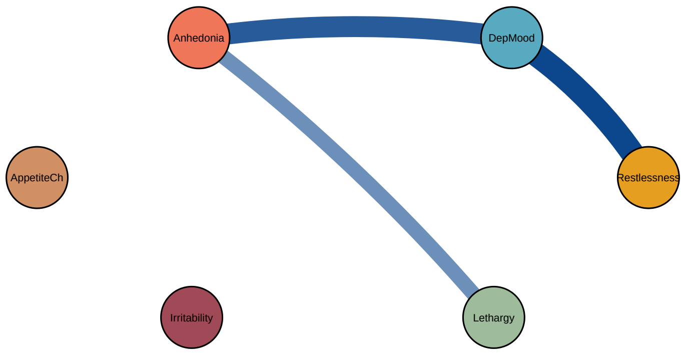

Symptom dynamics of MDD patients with IDS−SR score: 38

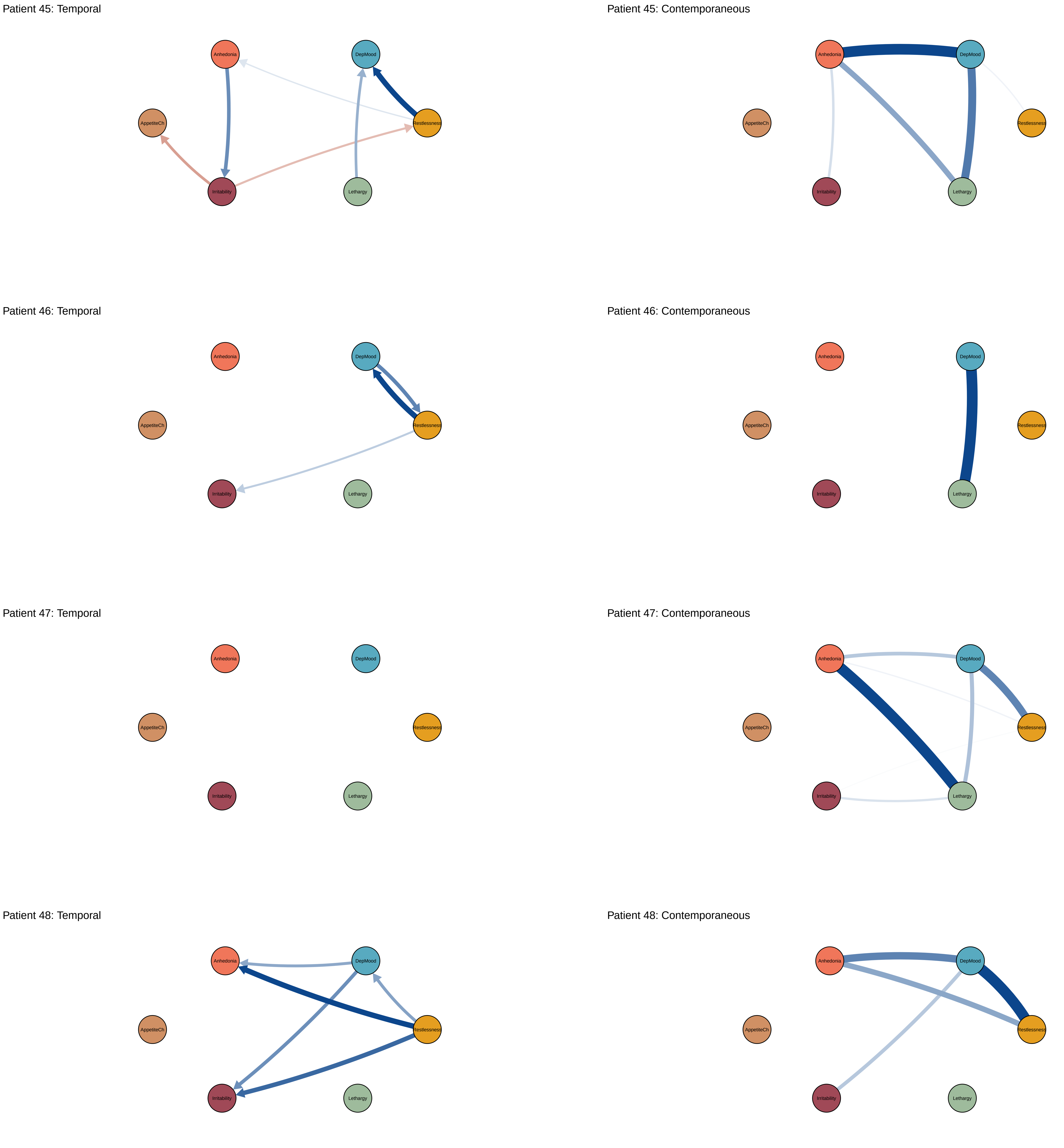

Symptom dynamics of MDD patients with IDS−SR score: 40

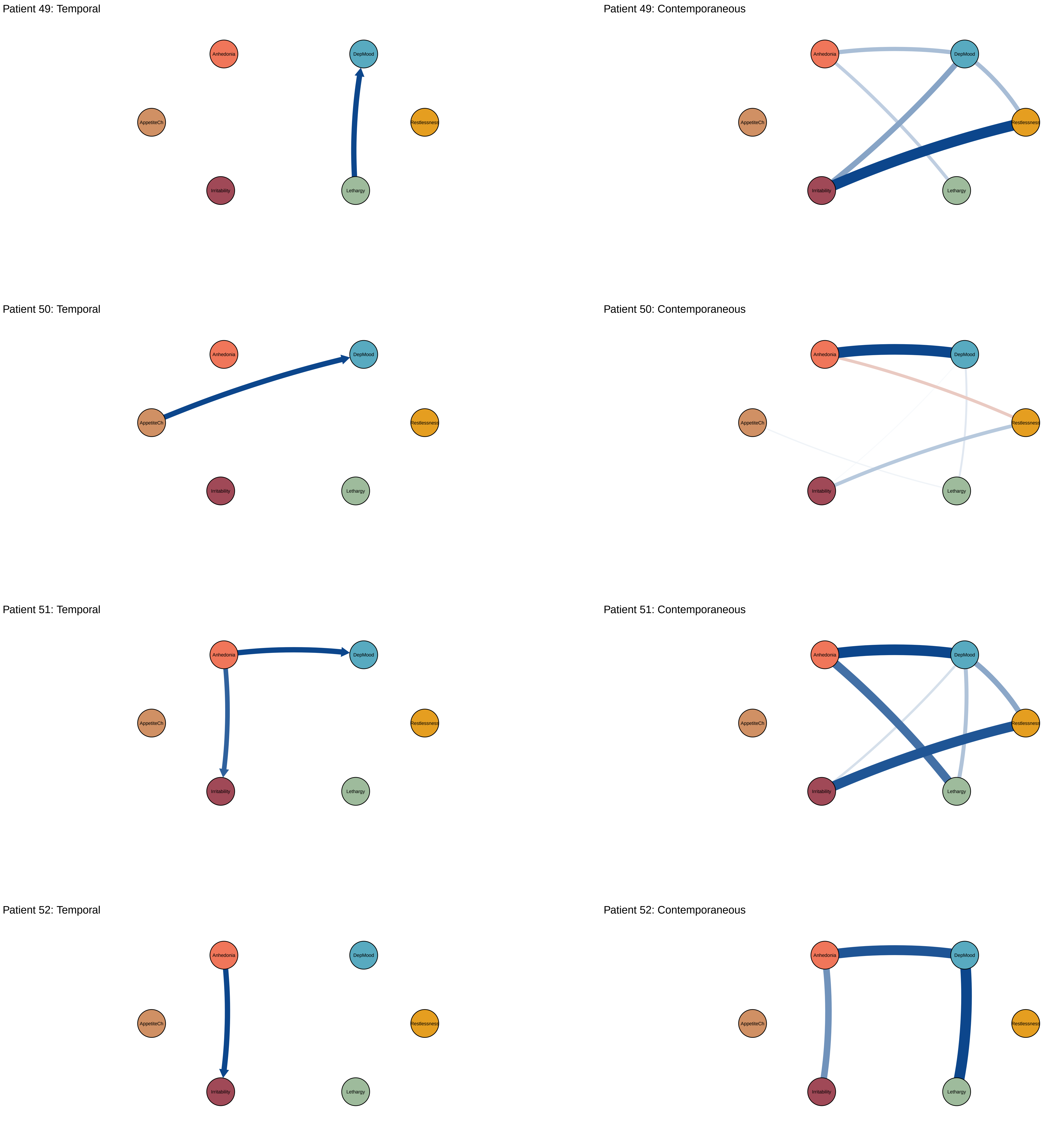

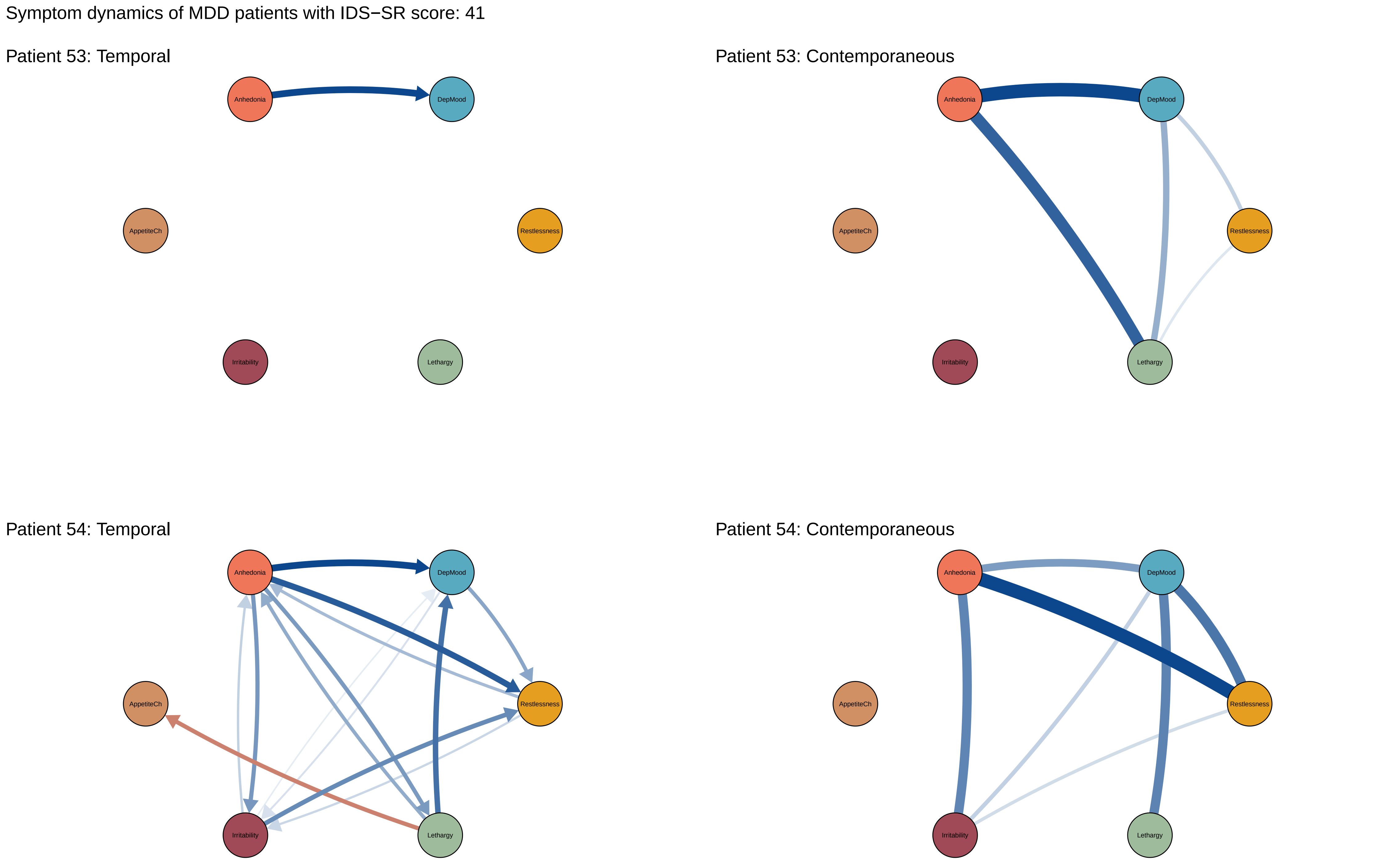
Symptom dynamics of MDD patients with IDS−SR score: 41
Patient 53: Temporal
Patient 53: Contemporaneous
Patient 54: Temporal
Patient 54: Contemporaneous
Anhedonia
DepMood
AppetiteCh
Restlessness
Irritability
Lethargy

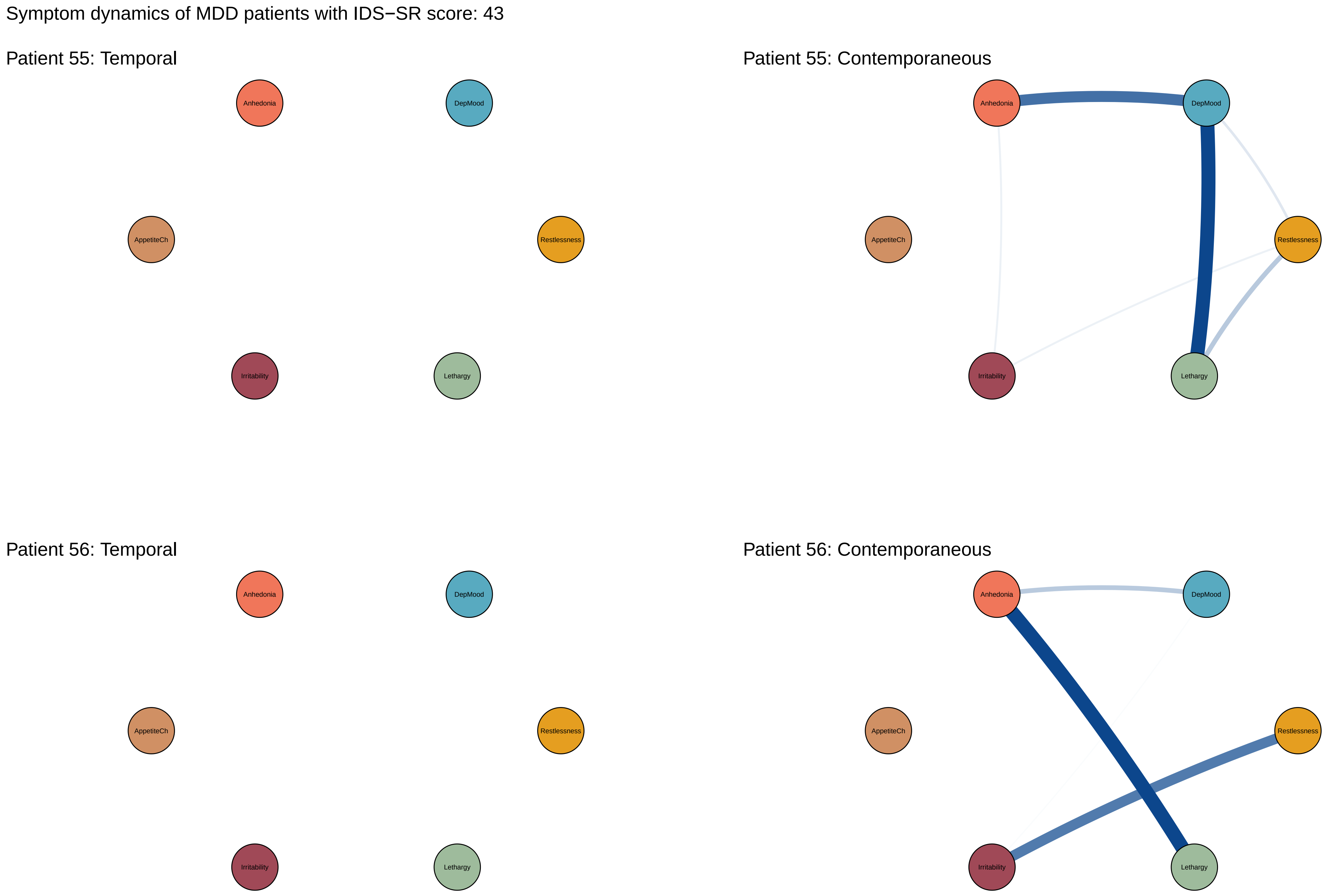
Symptom dynamics of MDD patients with IDS−SR score: 43
Patient 55: Temporal
Anhedonia
DepMood
AppetiteCh
Restlessness
Irritability
Lethargy
Patient 55: Contemporaneous
Anhedonia
DepMood
AppetiteCh
Restlessness
Irritability
Lethargy
Patient 56: Temporal
Anhedonia
DepMood
AppetiteCh
Restlessness
Irritability
Lethargy
Patient 56: Contemporaneous
Anhedonia
DepMood
AppetiteCh
Restlessness
Irritability
Lethargy

Symptom dynamics of MDD patients with IDS−SR score: 44

Patient 57: Temporal

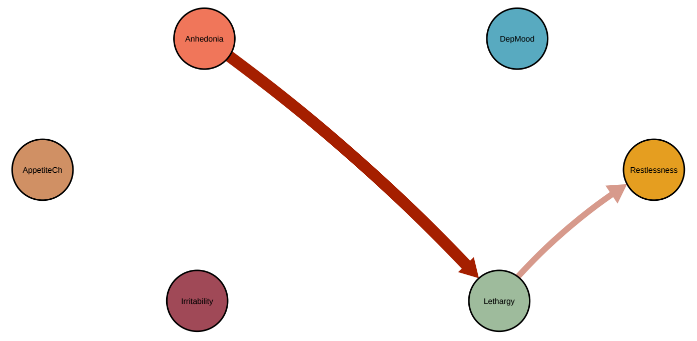

Patient 57: Contemporaneous

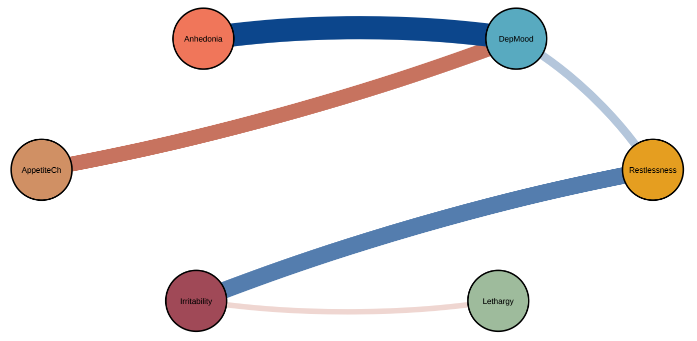

Patient 58: Temporal

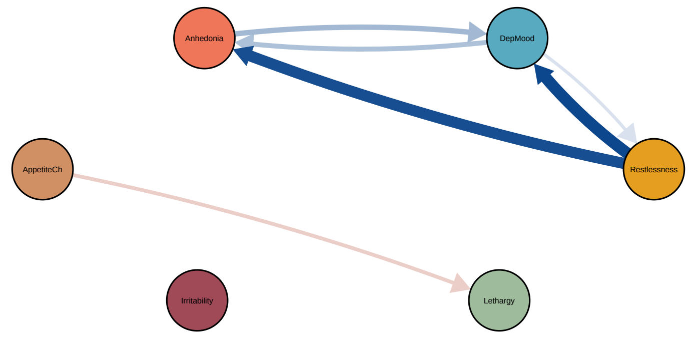

Patient 58: Contemporaneous

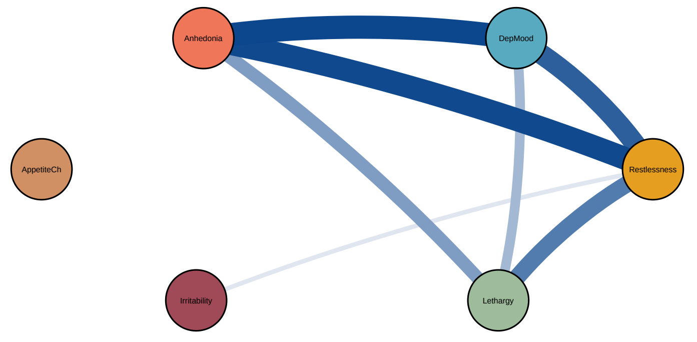

Patient 59: Temporal

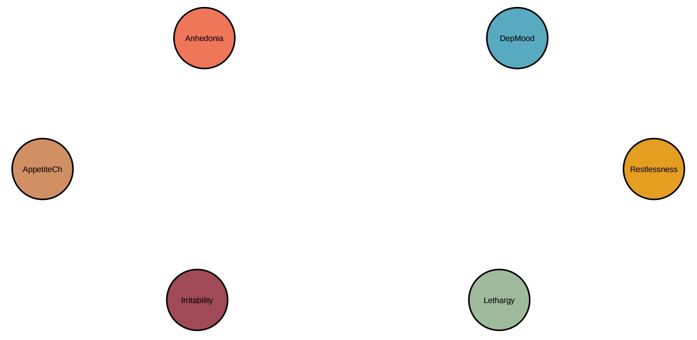

Patient 59: Contemporaneous

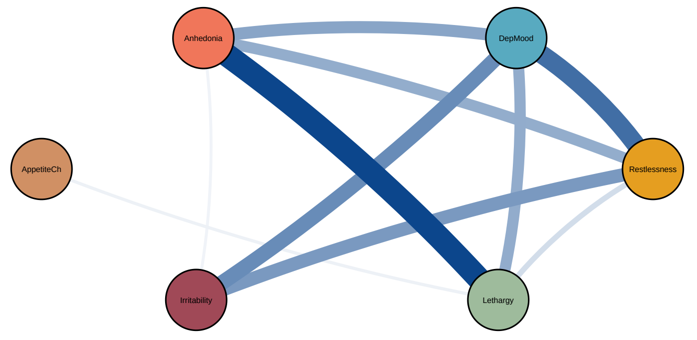

Patient 60: Temporal

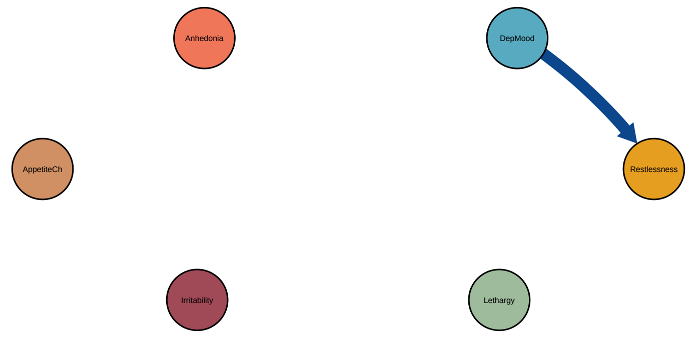

Patient 60: Contemporaneous

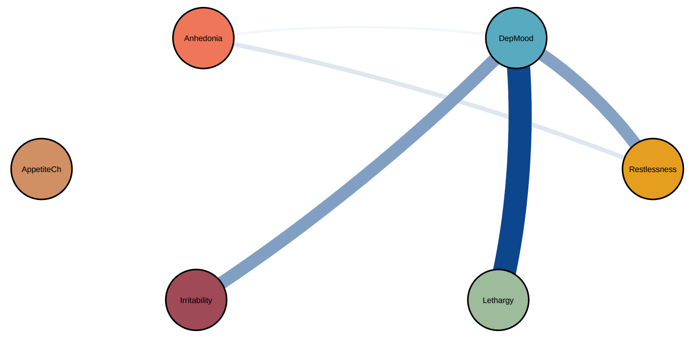

Patient 61: Temporal

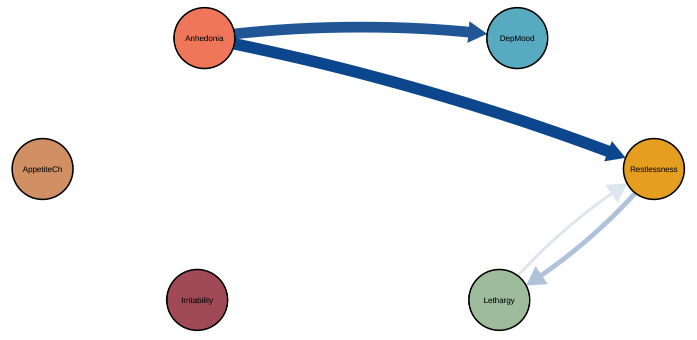

Patient 61: Contemporaneous

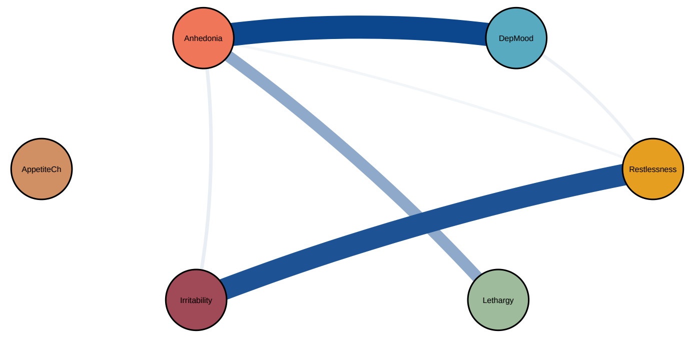

Patient 62: Temporal

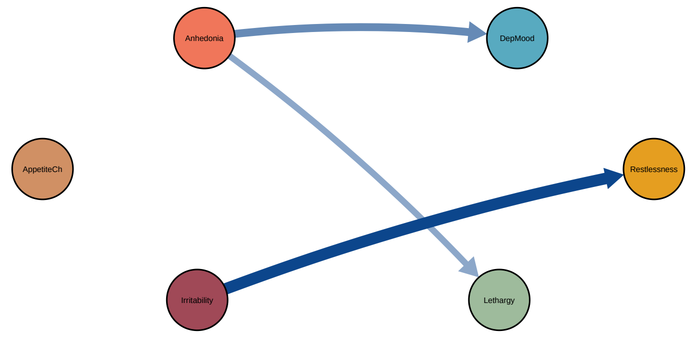

Patient 62: Contemporaneous

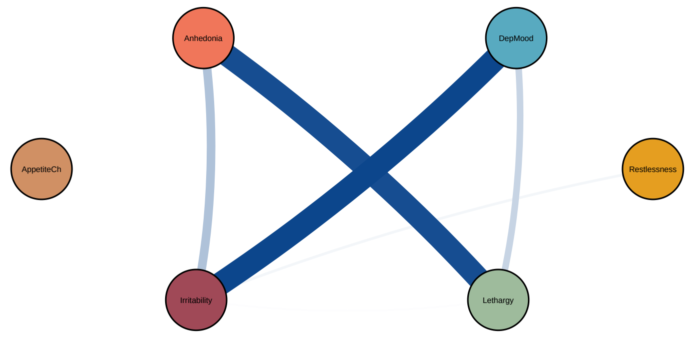

Symptom dynamics of MDD patients with IDS−SR score: 45

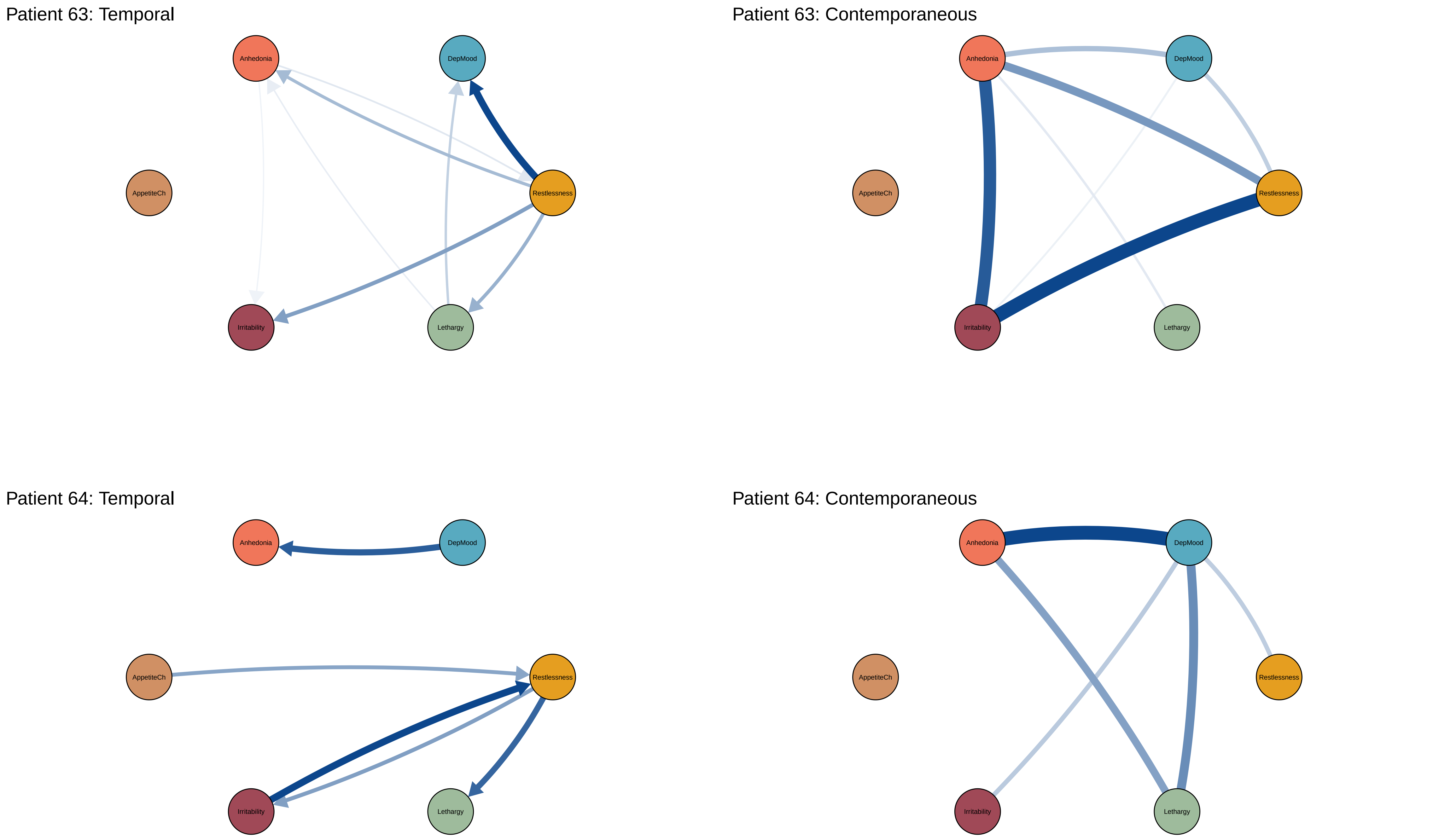

Symptom dynamics of MDD patients with IDS−SR score: 46

Patient 65: Temporal

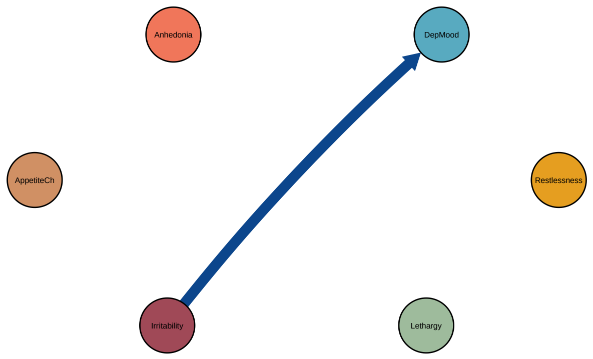

Patient 65: Contemporaneous

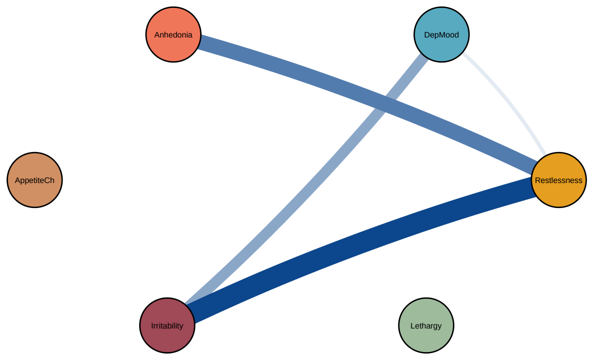

Patient 66: Temporal

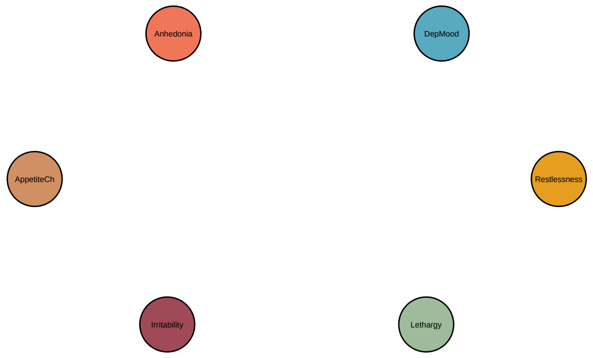

Patient 66: Contemporaneous

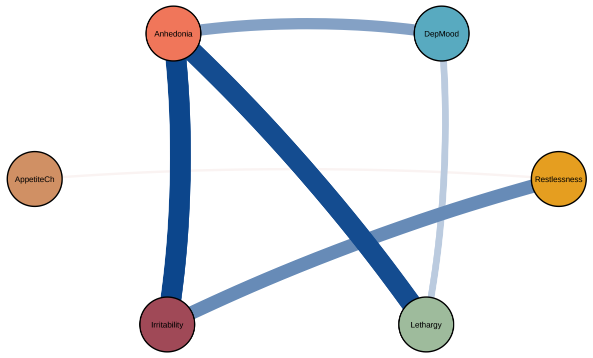

Patient 67: Temporal

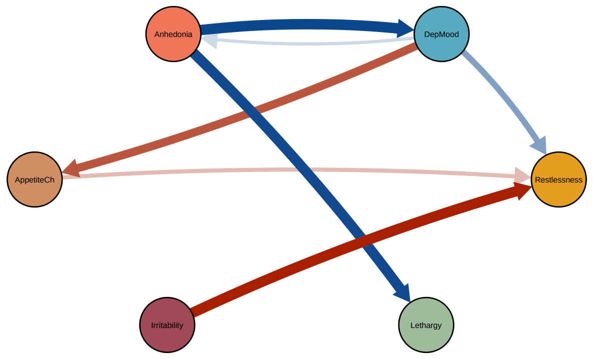

Patient 67: Contemporaneous

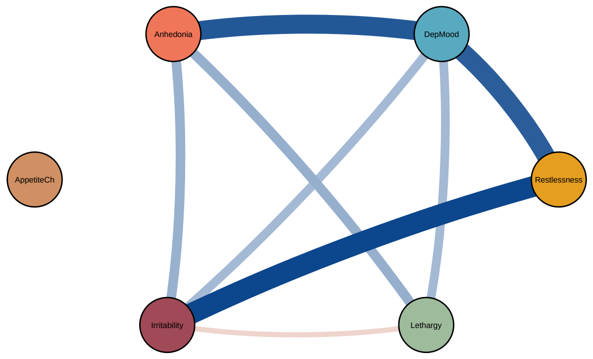

Symptom dynamics of MDD patients with IDS−SR score: 47

Patient 68: Temporal

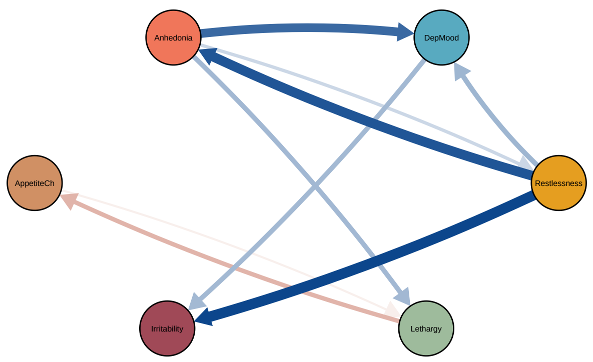

Patient 68: Contemporaneous

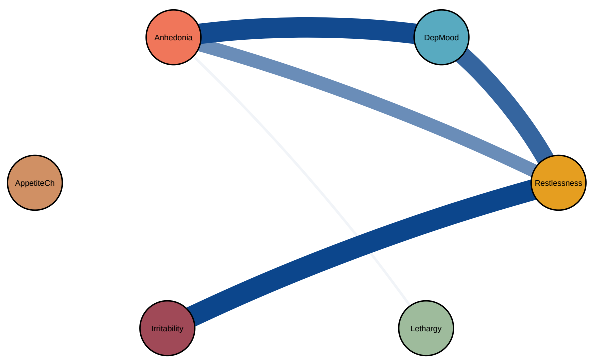

Patient 69: Temporal

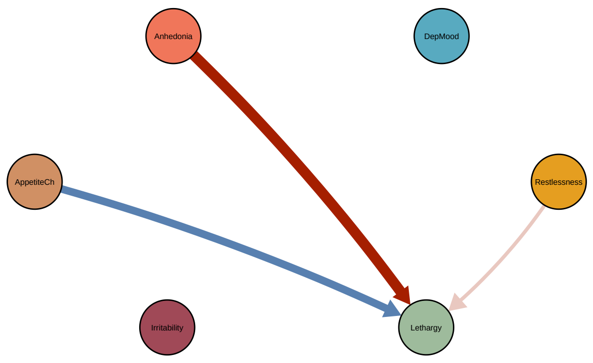

Patient 69: Contemporaneous

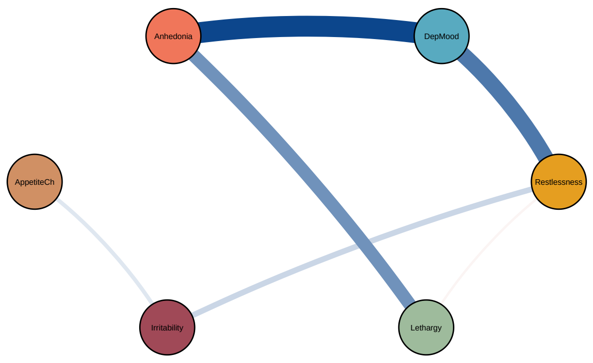

Patient 70: Temporal

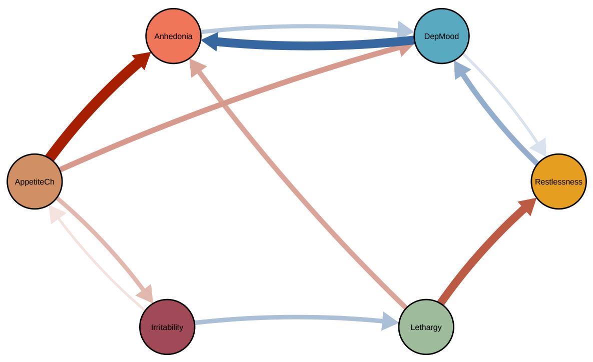

Patient 70: Contemporaneous

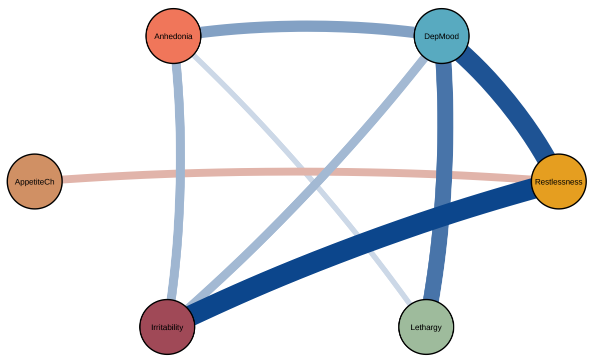

Symptom dynamics of MDD patients with IDS−SR score: 49

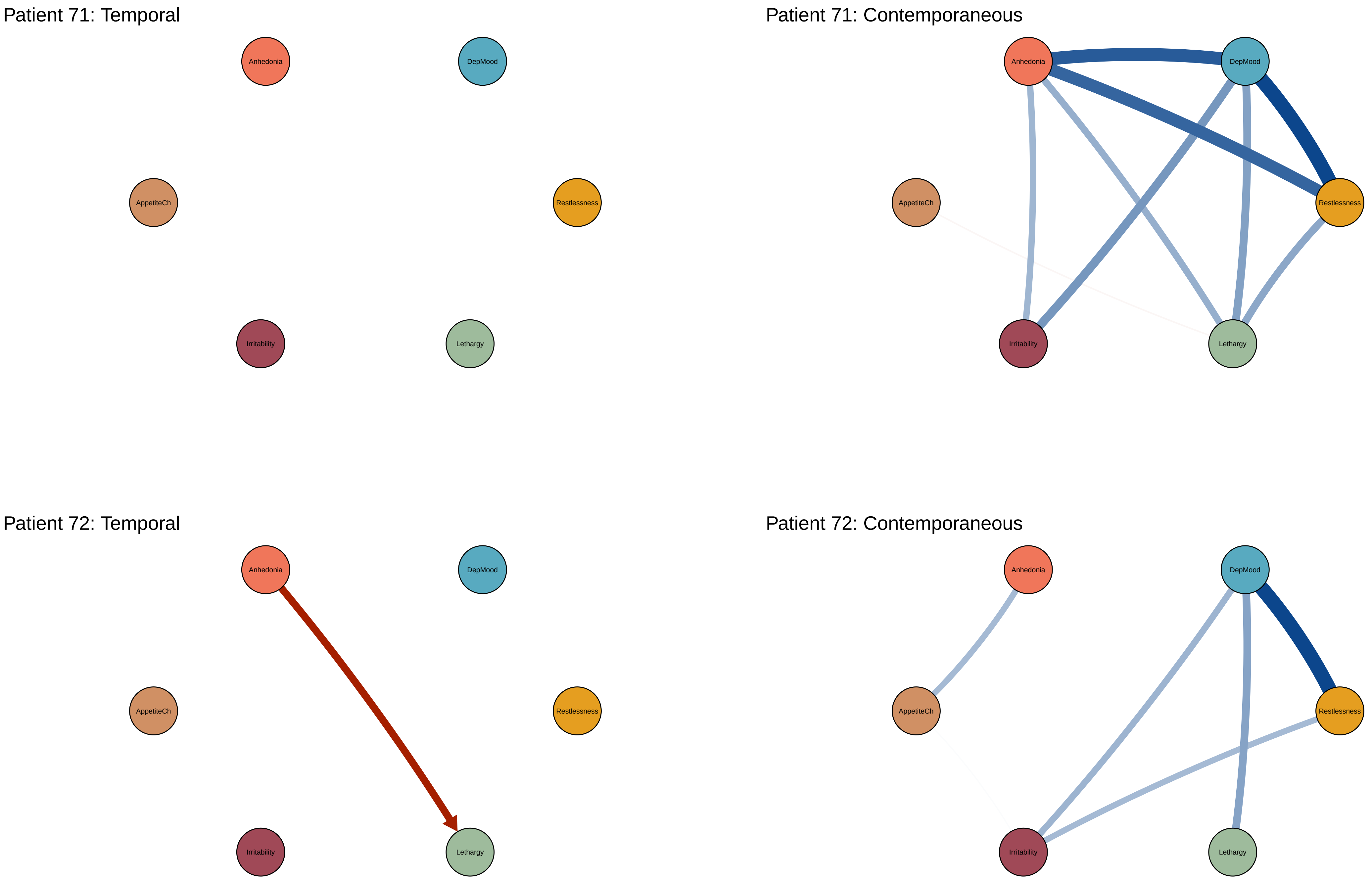

Symptom dynamics of MDD patients with IDS−SR score: 51

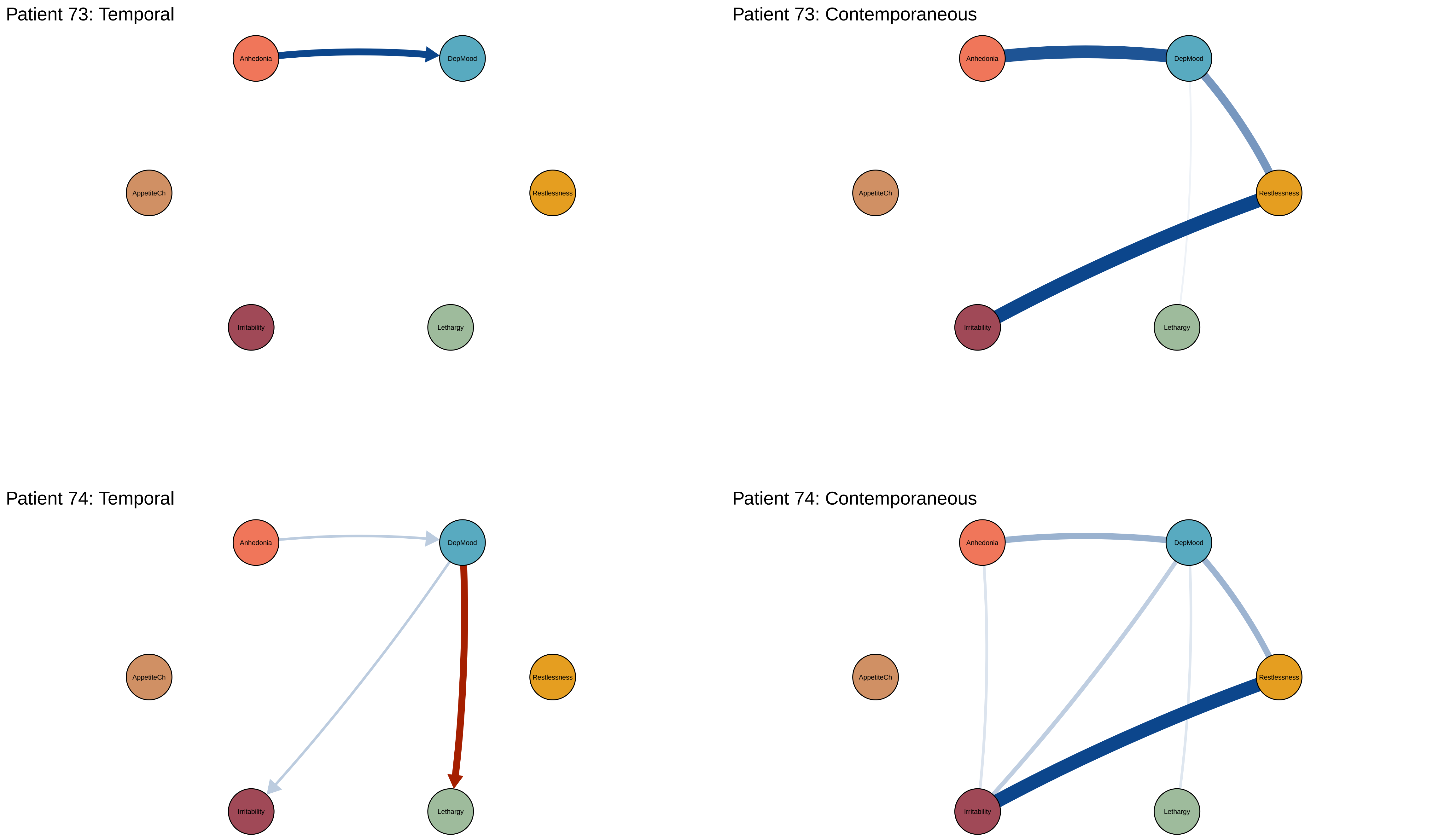

Supplement: Ebrahimi et al. supplementary material 2 — Ebrahimi et al. supplementary material [file S0007125024000199sup002.pdf]
