# Supplementary material for: Towards precision in the diagnostic profiling of patients: leveraging symptom dynamics as a clinical characterisation dimension in the assessment of major depressive disorder
Source: Br J Psychiatry. 2024 May;224(5):157–63. doi: 10.1192/bjp.2024.19 (PMC11039556; doi:10.1192/bjp.2024.19)
Supplement: Ebrahimi et al. supplementary material 3 — Ebrahimi et al. supplementary material [file S0007125024000199sup003.pdf]

Supplemental File 3: Ecological Momentary Assessment Items

How are you feeling at this moment?

Depressed mood
I feel down
I feel cheerful (reversed)

Anhedonia
I feel indifferent
I feel listless
I feel enthusiastic (reversed)

Appetite change
I am hungry

Restlessness
I feel calm (reversed)
I feel stressed
I feel relaxed (reversed)

Irritability
I feel irritated

Lethargy
I am tired
I feel energetic (reversed)

Visual analogue scale
0 (Not at all) -- 100 (Very much)
